# The novel family of Warbicin^®^ compounds inhibits glucose uptake both in yeast and human cells and restrains cancer cell proliferation

**DOI:** 10.3389/fonc.2024.1411983

**Published:** 2024-08-22

**Authors:** Ward Vanthienen, Juan Fernández-García, Maria Francesca Baietti, Elisa Claeys, Frederik Van Leemputte, Long Nguyen, Vera Goossens, Quinten Deparis, Dorien Broekaert, Sophie Vlayen, Dominique Audenaert, Michel Delforge, Alessandro D’Amuri, Griet Van Zeebroeck, Eleonora Leucci, Sarah-Maria Fendt, Johan M. Thevelein

**Affiliations:** ^1^ Center for Microbiology, VIB, Leuven-Heverlee, Belgium; ^2^ Laboratory of Molecular Cell Biology, Institute of Botany and Microbiology, KU Leuven, Leuven-Heverlee, Belgium; ^3^ VIB-KU Leuven Center for Cancer Biology, Leuven, Belgium; ^4^ Laboratory of Cellular Metabolism and Metabolic Regulation, Department of Oncology, KU Leuven and Leuven Cancer Institute (LKI), Leuven, Belgium; ^5^ TRACE PDX Platform, Laboratory of RNA Cancer Biology, LKI Leuven Cancer Institute, KU Leuven, Leuven, Belgium; ^6^ Screening Core, VIB, Ghent, Belgium; ^7^ Centre for Bioassay Development and Screening (C-BIOS), Ghent University, Ghent, Belgium; ^8^ LKI Leuven Cancer Institute Leuven, KU Leuven, Leuven, Belgium; ^9^ Anatomic Pathology Unit, ‘A. Perrino’ Hospital, Brindisi, Italy; ^10^ NovelYeast bv, Bio-Incubator, BIO4, Leuven-Heverlee, Belgium

**Keywords:** glucose uptake, yeast, cancer cells, Warburg effect, transport-associated phosphorylation, synthetic inhibitors, Warbicin^®^

## Abstract

Many cancer cells share with yeast a preference for fermentation over respiration, which is associated with overactive glucose uptake and breakdown, a phenomenon called the Warburg effect in cancer cells. The yeast *tps1Δ* mutant shows even more pronounced hyperactive glucose uptake and phosphorylation causing glycolysis to stall at GAPDH, initiation of apoptosis through overactivation of Ras and absence of growth on glucose. The goal of the present work was to use the yeast *tps1Δ* strain to screen for novel compounds that would preferentially inhibit overactive glucose influx into glycolysis, while maintaining basal glucose catabolism. This is based on the assumption that the overactive glucose catabolism of the *tps1Δ* strain might have a similar molecular cause as the Warburg effect in cancer cells. We have isolated Warbicin**^®^** A as a compound restoring growth on glucose of the yeast *tps1Δ* mutant, showed that it inhibits the proliferation of cancer cells and isolated structural analogs by screening directly for cancer cell inhibition. The Warbicin**^®^** compounds are the first drugs that inhibit glucose uptake by both yeast Hxt and mammalian GLUT carriers. Specific concentrations did not evoke any major toxicity in mice but increase the amount of adipose tissue likely due to reduced systemic glucose uptake. Surprisingly, Warbicin**^®^** A inhibition of yeast sugar uptake depends on sugar phosphorylation, suggesting transport-associated phosphorylation as a target. *In vivo* and *in vitro* evidence confirms physical interaction between yeast Hxt7 and hexokinase. We suggest that reversible transport-associated phosphorylation by hexokinase controls the rate of glucose uptake through hydrolysis of the inhibitory ATP molecule in the cytosolic domain of glucose carriers and that in yeast *tps1Δ* cells and cancer cells reversibility is compromised, causing constitutively hyperactive glucose uptake and phosphorylation. Based on their chemical structure and properties, we suggest that Warbicin**^®^** compounds replace the inhibitory ATP molecule in the cytosolic domain of the glucose carriers, preventing hexokinase to cause hyperactive glucose uptake and catabolism.

## Introduction

Cells of many cancer types share with yeast cells a preference for fermentation over respiration of glucose even under aerobic conditions and also in cancer types that have maintained the same full respiratory capacity as present in non-cancer cells. Both in cancer cells and in yeast, this fermentative metabolism is correlated with rapid growth and proliferation (1, 2). In cancer cells this phenomenon is called the Warburg effect (3) and leads to lactic acid production. In yeast, the Crabtree effect causes glucose repression of mitochondrial respiration, which triggers ethanol production under aerobic conditions (4). However, in yeast mutants lacking glucose repression of respiration, ethanol fermentation is still present, which creates a similar situation as with the Warburg effect in cancer cells (5). Although the Warburg effect has been studied extensively, the primary biochemical cause responsible for the overactive glycolytic flux remains uncertain (6). A general property of cancer cells is hyperactive glucose uptake which forms the basis for detection of a wide variety of tumor types using 2-^18^F-fluoro-2-deoxyglucose and positron emission tomography (7). Glucose uptake and phosphorylation are also considered to exert major control on glycolytic flux (8–11). Overexpression of glucose transporters and/or hexokinase is a common feature of many cancer types (11–16). Many studies have provided evidence that the Warburg effect is important for the rapid proliferation and survival of cancer cells (17). Hence, the Warburg effect appears to be a highly promising target for anti-cancer therapies, further supported by its widespread prevalence in cancer cells and its correlation with the aggressiveness of tumors (10, 18, 19).

The yeast *Saccharomyces cerevisiae* is well known for its high capacity of alcoholic fermentation, being exploited for production of wine, beer and other alcoholic beverages. When exposed to glucose or other related fermentable sugars, it rapidly represses respiration activity at transcriptional and post-translational levels and fully switches to ethanol fermentation, even under fully aerobic conditions (20). In addition, insufficient respiratory capacity results in short-term ‘overflow’ metabolism at the level of pyruvate (21). Although multiple crucial genetic components of the glucose repression pathway have been identified, the initial glucose sensing mechanism, in particular the involvement of the major hexokinase of yeast, Hxk2, and the interplay with the glucose sensing mechanisms for activation of the cAMP-protein kinase A (PKA) pathway are not well understood (22, 23). It is also unclear whether the physiological resemblance between the preference of yeast and cancer cells for fermentation is mimicked at the molecular level by common underlying mechanisms (1). On the other hand, a common mechanism between yeast and cancer cells is the activation of Ras by the glycolytic intermediate fructose-1,6-bisphosphate (Fru1,6bisP), which in both cases may couple increased glycolytic flux to stimulation of cell growth and proliferation (24).

Glucose is taken up in mammalian and yeast cells by low- and high-affinity facilitated diffusion carriers belonging to the Major Facilitator Superfamily (25–28). Mammalian GLUT carriers and yeast Hxt carriers share a similar structure with 12 transmembrane domains, cytoplasmic N- and C-termini and they have significant sequence similarity. The presence of a cytosolic ATP-binding domain in the mammalian GLUT1 carrier and its role in inhibition by ATP of glucose uptake has been documented in detail (29–33) and a similar mechanism has been suggested for GLUT4 (34, 35). The presence and possible role of ATP-binding in yeast Hxts has not been investigated. Several inhibitors of the mammalian GLUT carriers are available and inhibit cancer cell proliferation (36). Little is known about their effect on yeast Hxts.

After its uptake, glucose is phosphorylated in mammalian and yeast cells by hexokinase enzymes, which also belong to the same family and show significant sequence similarity (37). There is a conspicuous difference between the yeast and mammalian hexokinases with respect to their control by feedback inhibition. Whereas mammalian hexokinases are controlled through feedback-inhibition by their product glucose-6-phosphate (Glu6P), this is not the case for yeast hexokinase (11, 37, 38). The equivalent control has remained elusive until the unexpected discovery that a series of yeast mutants with varying deficiencies for growth on glucose or related fermentable sugars, all carried mutations in *TPS1*, the gene encoding trehalose-6-phosphate (Tre6P) synthase, the first enzyme of trehalose biosynthesis (39–42). This led to the discovery that Tre6P, made from Glu6P and UDPG, acts as feedback inhibitor of yeast hexokinase (38). While the *tps1* mutants are highly sensitive to even low mM concentrations of glucose (24, 43), deletion of *HXK2*, encoding the most active hexokinase isoenzyme, restores normal growth on glucose (44). Addition of glucose to *tps1Δ* cells causes dramatic hyperaccumulation of sugar phosphates, especially Fru1,6bisP, and depletion of ATP and downstream glycolytic metabolites (40, 41). Recent work has provided evidence that the hyperaccumulation of Fru1,6bisP is, at least in part, due to persistent glucose-induced intracellular acidification, which likely compromises glyceraldehyde-3-phosphate dehydrogenase (GAPDH) activity because of its unusually high pH optimum (45).

Obligatory coupling of sugar transport and phosphorylation is well known from the bacterial phosphotransferase system (PTS) (46). Although a similar system has not been found in eukaryotic cells, evidence has been reported suggesting optional transport-associated (or vectorial) phosphorylation (TAP) of glucose both in yeast and mammalian cells. In yeast, 2-deoxyglucose pool labeling experiments consistently revealed that under the conditions of these experiments, 2-deoxyglucose entered the cells first in the 2-deoxyglucose-6-phosphate pool and only subsequently in the free 2-deoxyglucose pool (47–52). Although these papers suggested transport-associated phosphorylation of sugar, interpretation of the results has been controversial (53). The phenomenon received little further attention after the demonstration by a quench-flow technique that high-affinity uptake measured on a sub-second time scale was not dependent on presence of the sugar kinases and the conclusion that glucose entered yeast cells as free sugar (54). However, the anaerobic/buffer conditions used in the quench-flow experiments predict very low intracellular ATP levels, which may prevent effective transport-associated phosphorylation at very short time scales. In mammalian cells, transport-associated phosphorylation of 2-deoxyglucose was suggested to occur in adipocytes (55), Ehrlich ascites tumor cells (56) and Murine Sarcoma Virus-infected Mouse Embryo Cells (57). Physical binding of hexokinase to plasma membranes from glioma (58) and ascites tumor cells (59) and to GLUT4 in muscle cells (60) has also been documented, suggesting possible coupling between glucose uptake and subsequent intracellular phosphorylation.

Because of the importance of the Warburg effect for proliferation and viability of cancer cells, many studies have explored inhibition of glycolysis for anti-cancer therapy (61–66). Such drugs, however, generally suffer from adverse side effects because of the universal importance of glucose catabolism in virtually all cell types. Similar findings have been made with glucose uptake inhibitors (36, 67, 68).

In this work, we have used the yeast *tps1Δ* strain to screen for small molecules that counteract hyperactive glucose influx into glycolysis, while still allowing sufficient basal glucose metabolism, an essential requirement for anticancer drugs targeting the Warburg effect. This resulted in identification of the novel Warbicin**^®^** family of putative anticancer drugs, that inhibit glucose uptake both by yeast and mammalian glucose carriers. Warbicin**^®^** compounds counteract proliferation in a dose-dependent manner in different types of cancer cells. The new glucose uptake inhibitors provide potent tools to investigate the physiological role of the Warburg effect and its primary cause, including the possible role of transport-associated phosphorylation in view of their dependency in yeast on glucose phosphorylation.

## Materials and methods

### Cell line and plasmid overview

(i) *S. cerevisiae*

All *S. cerevisiae* strains used and constructed in this work shared the W303 genetic background (**Table 1**), with the exception of the *hxt^0^* strains with the RE700A and EBY.VW4000 backgrounds. Both gene deletion and plasmid transformation strategies were performed by following the Gietz heat shock protocol (70). For genomic deletions, either antibiotic resistance gene cassettes or auxotrophic markers were PCR amplified with primer tails homologous to the 50 bp upstream and downstream regions of the gene of interest. Using heat shock at 42°C for 30 min, cells were transformed for homologous recombination or for introduction of a plasmid. For antibiotic selection, cells were incubated in medium without selection for 4 h prior to plating, whereas for auxotrophic selection, cells were plated immediately after the transformation protocol.

**Table 1:** Overview of the *S. cerevisiae* yeast strains used and constructed in this work.

Strain	Main genotype	Complete genotype	Source/Reference
W303-1A	Wild type for all strains, except where indicated	*MAT*a *leu2-3,112 trp1-1 can1-100 ura3-1 ade2-1 his3-11,15 GAL SUC mal*	(69)
WVT2	*tps1Δ*	*tps1::KanMX*	This work
WVT27	*tps1Δ hxt2Δ*	*tps1::KanMX, hxt2::NatMX*	This work
WVT28	*tps1Δ hxt4Δ*	*tps1::KanMX, hxt4::NatMX*	This work
WVT29	*tps1Δ hxt5Δ*	*tps1::KanMX*, *hxt5::NatMX*	This work
WVT22	*tps1Δ hxt6, 7Δ*	*tps1::KanMX*, *hxt6, 7::NatMX*	This work
WVT24	*tps1Δ hxt6, 7, 4Δ*	*tps1::HphMX, hxt6, 7::NatMX, hxt4::KanMX*	This work
WVT25	*tps1Δ hxt6, 7, 5Δ*	*tps1::HphMX, hxt6, 7::NatMX, hxt5::KanMX*	This work
WVT23	*tps1Δ hxt6, 7, 2Δ*	*tps1::HphMX, hxt6, 7::NatMX, hxt2::KanMX*	This work
WVT35	*tps1Δ hxt6, 7, 2, 4Δ*	*tps1::KanMX, hxt6, 7::looped, hxt2::NatMX, hxt4::looped*	This work
WVT36	*tps1Δ hxt6, 7, 2, 4, 5Δ*	*tps1::HphMX, hxt6, 7::looped, hxt2::NatMX, hxt4::looped, hxt5::KanMX*	This work
WVT8	Hxt7-Citrine	*hxt7::HXT7-Citrine-KanMX*	This work
WVT9	Hxk2-Citrine	*hxk2::HXK2-Citrine-KanMX*	This work
WVT39	Hxk1-Citrine	*hxk1::HXK1-Citrine-KanMX*	This work
WVT40	Glk1-Citrine	*glk1::GLK1-Citrine-KanMX*	This work
WVT41	Hxt7-NCitr	*hxt7::HXT7-NCit-URA3*	This work
WVT11	Hxt7-NCitr Hxk2-CCitr	*hxt7::HXT7-NCit-URA3, hxk2::HXK2-CCit-HIS3*	This work
WVT42	Hxt7-NCitr Hxk1-CCitr	*hxt7::HXT7-NCit-URA3, hxk1::HXK1-CCit-HIS3*	This work
WVT43	Hxt7-NCitr Glk1-CCitr	*hxt7::HXT7-NCit-URA3, glk1::GLK1-CCit-HIS3*	This work
WVT46	*HXK2 hxk1Δ glk1Δ*	*hxk1::NatMX, glk1::KanMX*	This work
WVT47	*hxk2Δ HXK1 glk1Δ*	*hxk2::NatMX, glk1::KanMX*	This work
WVT48	*hxk2Δ hxk1 GLK1*	*hxk2::NatMX, hxk1::KanMX*	This work
WVT49	*hxk2Δ hxk1Δ glk1Δ*	*hxk2::HphMX, hxk1::NatMX, glk1::KanMX*	This work
JT20836	*hxk2Δ hxk1Δ glk1Δ tps1Δ*	*hxk2::TRP1*, *hxk1::HIS3*, *glk1::KanMX*, *tps1::TRP1*	This work
WVT50	*gal80Δ*	*gal80::NatMX*	This work
WVT51	*gal80Δ gal1Δ*	*gal80::NatMX, gal1::KanMX*	This work
WVT52	*gal80Δ gal3Δ*	*gal80::NatMX, gal3::KanMX*	This work
WVT53	*gal80Δ gal3Δ gal1Δ*	*gal80::NatMX, gal3::KanMX*, *gal1::HphMX*	This work
JT5312	RE700A *hxt^0^ gal2Δ*	RE700A *MAT*a *hxt1, 2, 3, 4, 5, 7Δ*, *gal2::URA3*	This work
WVT54	EBY.VW4000 *hxt^0^ erg4Δ*	EBY.VW4000 *leu2-3,112 ura3-52 trp1-289 his3Δ1 MAL2-8C SUC2 hxt17Δ hxt13Δ hxt15Δ hxt16Δ hxt14Δ hxt12Δ hxt9Δ hxt11Δ hxt10Δ hxt8Δ hxt1-7Δ gal2Δ slt1Δ agt1Δ ydl247wΔ yjr160cΔ*, *erg4::NatMX*	This work

(ii) Human cell lines

An overview of the human cell lines used in this work is shown in **Table 2**. Growth analysis of adherent epithelial cell lines was performed in the laboratory of Cellular Metabolism and Metabolic Regulation. The human cell lines were also cultivated in the L2 cell culture room in the Molecular Cell Biology laboratory of Prof. Patrick Van Dijck (KU Leuven, VIB) for glucose uptake studies.

**Table 2:** Overview of the human cell lines used in this work.

Cell line	Source or reference	Source
A549, lung adenocarcinoma (type II alveolar epithelium)	ATCC, CCL-185	Kindly provided by Prof. Sarah-Maria Fendt.
MCF10A, non-tumorigenic breast epithelial cell line	ATCC, CRL-10317	Kindly provided by Prof. Sarah-Maria Fendt.
MCF10A, non-tumorigenic breast epithelial cell line, transformed with *H-RAS^V12^*	(71)	Kindly provided by Prof. Sarah-Maria Fendt.
KMS-12-PE, multiple myeloma	German Collection of Microorganisms and Cell Cultures GmbH, ACC-606	Kindly provided by Prof. Michel Delforge.

(iii) Plasmids

Plasmids were constructed using Gibson Assembly^®^ cloning. Vectors of choice were always double digested and sticky ends were dephosphorylated by FastAP^™^. Inserts were PCR amplified by the Q5^®^ High-Fidelity polymerase and both the digested vectors and inserts were first gel-purified. Finally, Gibson cloning was performed by adding Gibson reagent in the recommended ratio of insert to vector. After incubation at 50°C for 30 min, the Gibson reaction mixture was transformed into TOP10 *E. coli* cells by heat shock. Positive clones were validated by PCR and sequence analysis. An overview of the plasmids used in this work is given in **Table 3**. For the cloning of *HXT7* and *HXK2*, DNA from the W303 genetic background was used as genomic template. The full-length *GLUT1* and *GLUT4* coding sequences were obtained from the UniProt database and were ordered as gBlocks^™^ from IDTechnologies. Additional point mutations were introduced by PCR amplification, using primers with tails designed to bear the intended mutations.

**Table 3:** Overview of the plasmids used and constructed in this work.

Plasmid	Plasmid backbone	Vector elements	Selection marker	Insert	Source
pYX112-HXT7-HA	pYX112	*TPI1* prom., low-copy	*URA3*	*HXT7-HA*	This work
pYX122(-Empty)	pYX122	*TPI1* prom., low-copy	*HIS3*	Empty	MCB Lab, KU Leuven
pYX122-Citrine				*Citrine*	This work
pYX122-CCitr				*CCitr*	This work
pFL38Kan-HXT7-HA	pFL38-Kan	*TEF1* prom., low-copy	*KanMX*	*HXT7-HA*	This work
pFL38Kan-GLUT1^V69M^				*GLUT1^V69M^-HA*	This work
pFL38Kan-GLUT4^V85M^				*GLUT4^V85M^-HA*	This work
pYC33(-Empty)	YCpLAC33	Low-copy	*URA3*	Empty	MCB Lab, KU Leuven
pYC33-HXK2				*HXK2*	This work
pYC33-HXK2-Citrine				*HXK2-Citrine*	This work
pYC33-NLS-HXK2				*NLS-HXK2*	This work
pYC33-NLS-HXK2-Citrine				*NLS-HXK2-Citrine*	This work
pGEX-GST	pGEX4T-1	Tac prom.	*Amp*	*GST*	GE Healthcare
pGEX-GST-HXK2				*GST-HXK2*	This work

### General growth conditions

(i) *E. coli*

*E. coli* cells were always cultivated in Luria broth (LB) medium at 37°C. For plasmid retention and propagation in TOP10 *E. coli* cells, 100 µg/mL ampicillin was added to liquid medium or solid plates. For protein production, BL21 *E. coli* cells were grown to exponential phase at 37°C and shifted to 20°C overnight after isopropyl β-D-1-thiogalactopyranoside induction (IPTG). Cells were made heat shock competent by rubidium chloride (72) after which vials were stored at -80°C for up to one year.

(ii) *S. cerevisiae*

Cells of *S. cerevisiae* were either grown in minimal or rich medium. For minimal medium, cells were grown in Complete Synthetic medium (MP biomedicals) containing 0.5% (w/v) ammonium sulphate (Sigma-Aldrich), 0.17% (w/v) yeast nitrogen base without amino acids and without ammonium sulphate, supplemented with 100 mg/L adenine. In the case of auxotrophic selection, the appropriate composition of essential amino acids (MP Biomedicals) was chosen. For liquid medium, pH was adjusted to 5.5 and for solid medium (2% agar) to 6.5 with KOH. For rich medium, cells were grown on YP medium (1% w/v yeast extract, 2% w/v bacteriological peptone), supplemented with 100 mg/L adenine. Cells of the EBY.VW4000 *hxt^0^* strain expressing a single HXT carrier or GLUT isoform were (pre)grown in YP medium with 58 mM maltose (which is taken up by its own dedicated maltose transporter). *S. cerevisiae* cells were always grown at 30°C. Liquid cultures were shaken at 200 rpm.

(iii) Human cell lines

The A549 cell line was always propagated in RPMI-1640 (Gibco^®^) medium containing 10 mM glucose. Medium was supplemented with heat-inactivated 10% Fetal Bovine Serum (FBS) and 1% (50 units/mL) Penicillin/Streptomycin (Pen/Strep, Thermo Fisher Scientific). A549 cells were passaged close to 80 - 90% confluency. MCF10A *H-RAS^V12^* cells were cultured in DMEM/F12 (Gibco^®^) medium supplemented with 5% horse serum, 1% Pen/Strep, 10 µg/mL insulin, 0.5 µg/mL hydrocortisone, 100 ng/mL cholera toxin and 20 ng/mL recombinant human EGF (73). MCF10A *H-RAS^V12^* cells were always passaged before reaching 50% confluency. The KMS-12-PE cell line was cultured in RPMI medium containing 10 mM glucose, 20% heat-inactivated FBS and 1% Pen/Strep. Since these cells grow in suspension, cell density was kept between 10^5^ and 10^6^ cells/mL. For assay purposes with varying glucose concentrations, appropriate amounts of medium containing glucose or no glucose were mixed to achieve the intended composition. Finally, all medium solutions were filter-sterilized prior to use. For passaging, cells were always washed first with dPBS (Gibco^®^), and for adherent cells, detached by Trypsin-EDTA (Gibco™) digestion for 5 min. Cells were always incubated at 37°C in the presence of 5% CO_2_.

### Compound libraries, ID and handling

The 40,000 compounds collection that we screened for restoration of growth of the yeast *tps1Δ* strain in the presence of 5 mM glucose, was comprised of three sets of chemical libraries: Chembridge Diverset, Enamine DLS set and Life Chemical Diversity set. This collection offers high structural diversity and a broad coverage of pharmacophore space, while also ensuring suitable physiochemical properties and absence of chemical groups with toxic properties. The compounds were dissolved in 1% DMSO (final concentration) and screened in a concentration of 50 µM in Yeast extract/Peptone medium with 100 mg/L adenine. Screening was performed in 96-well plates at 30°C with continuous shaking and evaluation of growth was performed after 24h by OD_600_ spectrophotometric quantification. Re-evaluation of the 640 hits obtained in the first screen led to 17 hits in the second screen, using the same threshold of growth in the presence of 5 mM glucose, of which finally only one compound could be confirmed consistently in subsequent tests and also showed a clear dose-dependent relationship.

WBC-A and the complete structural analog library (**Supplementary Table 2**) were purchased from different vendors, i.e. Enamine (n = 185), Life Chemicals (n = 10), LabNetwork (n = 4) and Uorsy (n = 4). WBC compounds in general are very insoluble in aqueous solutions, challenging their solubilization. Throughout this work, WBC compounds were always stock aliquoted at 50 mM concentration in 100% DMSO. In many cases, gentle sonication and heating up to 37°C was necessary to completely solubilize the compound stocks. Whereas WBC-A, -15C, -4C and -11C could be dissolved at 50 mM, some structural analogs remained in smooth or rough suspension, regardless of the concentration. Even though it is recommended to aliquot compound stocks, no significant decrease in compound strength by repeated freeze and thaw cycles in DMSO was observed.

### Assessment of cell proliferation and viability

#### Spot dilution assays for assessment of yeast growth

Growth of yeast in the presence of increasing glucose concentrations was determined on solid agar medium using spot assays with serial dilutions of the inoculum. The remaining yeast growth at the different dilutions, scored by visual inspection, revealed the level of sensitivity of the yeast strain to increasing concentrations of glucose in the medium. For determination of *tps1Δ* (*hxtΔ*) glucose sensitivity and the restoration of *hxk2Δ hxk1Δ glk1Δ* glucose growth, cells were always pregrown on Complete Synthetic medium containing 325 mM glycerol. For the *tps1Δ* (*hxtΔ*) spot assay, 325 mM glycerol was added in all the plates. For the assessment of *hxk2Δ hxk1Δ glk1Δ tps1Δ* glucose sensitivity, cells were pregrown on 110 mM galactose which was also added as a base carbon source in all plates. After pregrowth overnight, cells were harvested, washed with medium without sugar and resuspended to an OD of 0.25. Cells were spotted in five-fold dilution in 3 µL droplets each. Plates were allowed to dry after which they were incubated at 30°C for 2 to 3 days and images were taken.

#### Growth curves

Growth curves were measured based on the increase in OD of both yeast and human cell lines to assess the effect of Warbicin**^®^** compounds on general growth of the respective cell line.

(i)*S. cerevisiae*

Growth curves were made by measuring OD_595_ in a 96-well plate using the Multiskan^™^ FC Microplate Photometer (Thermo Fisher Scientific). Typically, for *tps1Δ* rescue experiments, cells were (pre)grown in Complete Synthetic medium containing 110 mM galactose. Compounds were always added 10 min prior to glucose addition to ensure maximal interaction between the cells and compounds. Cells originating from the preculture were harvested, washed in medium without sugar, after which they were resuspended to an OD of 0.1 in 200 µL of the appropriate medium. At least three technical replicates were included per condition. OD_595_ was measured at 30°C every 30 min with a 10-min shaking interval. For all growth curve experiments using *S. cerevisiae*, DMSO had a final concentration of 1%.

(ii) Human cell lines

For both adherent A549 and MCF10A *H-RAS^V12^* cell lines, growth curves were established using the IncuCyte^®^ ZOOM technology. By collecting phase contrast images, the IncuCyte^®^ software provides a calculated confluency percentage from which growth curves can be deducted. The cell number/density was measured by calculating the confluency of phase objects (cells) sticking on the bottom of the well. Cells were seeded at a density of 1000 - 1500 cells/well in a Nunc-Edge^™^ 96-well plate (Thermo Fisher Scientific) and growth was typically measured over 3 to 5 days. For the measurement of apoptosis induction using the MCF10A *H-RAS^V12^* cell line, the IncuCyte^®^ Caspase-3/7 green dye was added to the medium in the recommended concentration at the beginning of the growth curve experiment. Apoptosis was detected by taking fluorescent images by excitation at 488 nm followed by IncuCyte^®^ software analysis. Cell growth of the KMS-12-PE multiple myeloma cell line, which grows in suspension, was measured by manual cell counting. Cells were initially seeded at a density of 10^4^ cells/well and incubated for 4 days after which the cell number was counted. For all cell lines, the same medium used for pregrowth was also used for the growth curve experiment, albeit with different glucose concentrations depending on the experimental design. With compound administration, the final concentration of DMSO never exceeded 0.1%. Typically, at least four technical repeats were included per condition.

### Metabolite measurements in yeast

Metabolites were extracted from yeast cells and measured as described previously (24, 45). General methods will be discussed in brief.

(i) Sample collection

For measuring metabolite levels, yeast cells were typically grown in Complete Synthetic medium. To assess the effect of 10 mM glucose addition to *tps1Δ* and *tps1Δ hxt6,7,4,2,5Δ* cells, 325 mM glycerol was used as a carbon source. However, when comparing the effect of Warbicin**^®^** compounds on metabolite profiles of wild type and *tps1Δ* strains, cells were grown on 110 mM galactose. When grown to exponential phase, cells were harvested by centrifugation and washed twice with ice-cold 25 mM 2-(N-Morpholino)ethanesulfonic acid (MES) buffer, pH 6. Cells were suspended in Complete Synthetic medium without sugar at a concentration of 75 mg (wet weight)/mL followed by a temperature equilibration at 30°C in a shaking water bath for 30 min. Depending on the experiment, a final concentration of 2.5 mM, 7.5 mM or 10 mM glucose was added to the cell suspension. Compounds were added 10 min prior to glucose addition. At distinct time points, 1.5 mL cell suspension was quenched in 60% methanol at -40°C (74). Quenched samples were centrifuged at -10°C followed by aspiration of the supernatant and resuspension of the cell pellet in 0.5 mL 1 M HClO_4_. After mechanical lysis of the cell suspensions by glass beads, an additional volume of 0.5 mL 1 M HClO_4_ was added after which the samples were stored at -20°C.

(ii) Sample processing

After centrifugation of the yeast cell lysates at high speed, the supernatant fractions were collected. From here, lysate fractions were processed for neutralization. As such, to 250 µL cell lysate, 50 µL of 5 M K_2_CO_3_ was added, supplemented with 10 µL thymol blue (0.025%) to visually monitor the pH. After thorough mixing, samples were left to degas on ice for 15 min. Subsequently, samples were centrifuged at high speed from which 200 µL of the supernatant fraction was collected to which 100 µL 1 M HCl and 10 µL Tris-HCl (pH 7.5) was added. Samples were stored at -20°C.

(iii) Metabolite measurement

Through endpoint measurement of the absorbance of NADH or NADPH at 340 nm, metabolite concentrations in the yeast cells were calculated by applying Lambert’s Law. Different metabolites were measured through the use of coupled enzymatic reactions. In general, 50 µL of sample was incubated with 150 µL assay buffer (100 mM Tris-HCl, pH 7.5). Depending on the measured metabolite, different co-factors and auxiliary enzymes were added. For measuring glucose-6-phosphate, 1.07 mM NADP^+^ was added to the assay buffer after which the baseline absorbance was measured. The addition of 10 U/mL Glu6P dehydrogenase (Sigma-Aldrich G7877) oxidizes Glu6P while producing an equal amount of NADPH. After stabilization of the OD_340_ spectra, ATP concentrations were measured by additionally adding 10 mM MgCl_2_ and 0.5 mM glucose to the assay buffer. To start the enzymatic consumption of ATP, 35 U/mL hexokinase (Sigma-Aldrich H6380) was added. Finally, for measuring fructose-1,6-bisphosphate levels, 50 µL of sample was incubated with 150 µL assay buffer, supplemented with 0.012 mM NADH, 100 U/mL triosephosphate isomerase (Sigma-Aldrich T2391) and 2.5 U/mL glycerol-3-phosphate dehydrogenase (Sigma-Aldrich G6880). When NADH absorption was stable, 1.6 U/mL aldolase (Sigma-Aldrich A2714) was added to start the reaction. For all measured metabolites, after stabilization of the OD_340_ spectra, the difference between the initial baseline value and the final absorbance after enzymatic conversion of the measured metabolite was used to determine its concentration. To express metabolite levels in terms of cytosolic concentration, an intracellular volume of 12 µL/mg protein was assumed.

### Determination of glucose uptake activity

#### Zero-*trans* uptake (The unidirectional flux into the cell at initial rate)

(i) *S. cerevisiae*

Zero-*trans* uptake of radioactively labeled [U-^14^C]glucose, [U-^14^C]fructose or [U-^14^C]galactose by *S. cerevisiae* cells was determined in accordance with previous studies (45, 75). Cells were grown on different carbon sources depending on the experiment. For measuring glucose uptake activity in the *tps1Δ* and *tps1Δ hxtΔ* strains, cells were grown on YP medium containing 325 mM glycerol (measurement of 10 s). Typically, for compound characterization and kinetic studies in wild type and *tps1Δ* strains, cells were (pre)grown on Complete Synthetic medium supplemented with 110 mM galactose (measurement of 10 s). In the experiments on the influence of hexokinase activity on the sugar uptake rate, the measurement duration was shortened to 5 s and cells were always grown on YP medium containing 325 mM glycerol and 430 mM ethanol. After harvesting, cells were washed twice with ice-cold 25 mM MES-buffer pH 6, and resuspended in their respective medium without sugar to a final concentration of 45 mg (wet weight)/mL. The amount of added tracer was estimated to give a response close to at least 1000 counts per min in order to adequately counter background noise. Cells were first preincubated for 10 min at 30°C with the compound to acclimate the cells to the temperature and to allow adequate interaction with the compound prior to uptake. Next, hexose sugar was mixed with the cell suspension, which was then incubated at 30°C for 5 or 10 s, depending on the experiment, after which the cells were rapidly filtered over a glass microfiber filter (Whatman GF/C) and washed three times with ice-cold dH_2_O. The loaded filter was transferred to a scintillation vial containing 3 mL liquid scintillation cocktail (Ultima-Flo M, Perkin Elmer) and counted using the Hidex 300 SL. Three blank measurements per strain were typically included to account for background signal, for which the cells were first quenched before adding the radioactive label.

(ii)*A549 adenocarcinoma*

Zero-*trans* glucose uptake in A549 cells was approximated by measuring the uptake of [1,2-^3^H]2-deoxyglucose. For this purpose, A549 cells were pregrown in RPMI medium containing 10 mM glucose in a 24-well plate to a cell density of around 100,000 cells/well. Prior to adding radioactive label, cells were gently washed twice in Krebs-Ringer-HEPES buffer (50 mM HEPES pH 7.4, 137 mM NaCl, 4.7 mM KCl, 1.85 CaCl_2_, 1.3 mM MgSO_4_ and 0.1% w/v BSA) at 30°C to remove any residual sugar. RPMI medium without sugar containing the compound intended for treatment, was added to the cells for 15 min at 37°C to allow adequate interaction of the cells with the compound. The uptake measurement was initiated by adding an equal volume of medium containing radiolabeled 2DG. After 3 - 4 min incubation at 37°C, medium was aspirated and cells were gently washed three times with ice-cold Krebs-Ringer-HEPES buffer. Cells were lysed by adding 200 µL of ice-cold 0.1 M NaOH solution and incubating the plate for 10 min at 37°C. Cell lysates were transferred to scintillation vials for subsequent scintillation counting. For blank measurements, cells were incubated with 50 µM Cytochalasin B prior to the uptake measurement.

#### Glucose consumption over a longer time period

##### A549 adenocarcinoma and KMS-12-PE multiple myeloma

The influence of WBC compounds on glucose consumption over a longer time period was studied in the A549 adenocarcinoma and KMS-12-PE multiple myeloma cell lines. As such, different parameters needed to be optimized depending on whether cells were adherent or in suspension. As such, A549 cells were incubated at 125,000 cells/well in a 24-well plate in 300 µL RPMI medium for 8 h. For the KMS-12-PE cell line, 100,000 cells/well were incubated in 100 µL RPMI medium in a 96-well plate for 8 h. Medium was collected, spun down and HPLC-analyzed for measuring glucose and lactate levels. Metabolite levels were corrected for cell number, which always varied little over the span of 8 h. For every condition, at least 4 technical repeats were included.

### Fluorescence microscopy of yeast cells

Using fluorescence microscopy, both the localization of individual proteins as well as BiFC interactions in yeast cells were studied by genomic C-terminal tagging of proteins of interest with full-length or split Citrine halves (NCitr: Citrine^AA1-154^; CCitr: Citrine^AA155-236^) or by plasmid-based expression of fluorescent fusion proteins. The mCitrine fluorophore has an excitation and emission maximum at 516 and 529 nm, respectively. Using the Olympus FluoView^™^ 1000 confocal laser microscope, cells were excited by the 515 nm laser line with DM458/515 and emission was monitored using the band pass BA535-565 filter set. Images were scanned at 8.0 µs/pixel combined with a 60x oil objective lens (Olympus UPlanSAPO, N.A. 1.35) together with a digital zoom of 5x. For fluorescence microscopy assays, yeast cells were typically (pre)grown on YP containing 325 mM glycerol and 430 mM ethanol. A small sample was taken from the mother culture, spun down at 2000 rpm and resuspended in a smaller volume to concentrate the cells. Next, 5 µL of cell suspension was applied to a glass slide and sealed by a coverslip after which the slide was allowed to settle for at least 5 - 10 min prior to visualization.

### Pulldown followed by Western blot analysis for yeast proteins

(i) Purification of yeast GST-Hxk2

For expression of GST and GST-Hxk2 in *E. coli* BL21, cells were grown in LB to exponential phase at 37°C and subsequently induced by 0.3 mM IPTG at room temperature overnight. Cell pellets were harvested and washed in ice-cold 25 mM MES-buffer pH 6. Next, cells were resuspended in lysis buffer (50 mM Tris-HCl pH 7.5, 150 mM NaCl, 1 mM EDTA, 2.5 mM MgCl_2_) for 30 min on ice containing 5 mg/mL lysozyme for cell wall digestion. After incubation, three additional volumes of lysis buffer were added containing 1% Triton X-100 and protease inhibitor cocktail (Roche) followed by three cycles of sonication with intermediate pauses on ice. Cell lysates were clarified by centrifugation at 10,000 rcf and incubated with Glutathione Sepharose^™^ 4B resin (GE Healthcare). As such, beads were incubated with cell lysate for 1 to 2 h on a roller drum at 4°C followed by three wash steps with lysis buffer containing 1% Triton X-100.

(ii) Pulldown of yeast Hxt7-HA

Wild type yeast cells transformed with p*HXT7-HA* were grown on uracil-deficient medium containing 110 mM galactose until exponential phase. Cells were harvested, washed with 25 mM MES pH 6, and resuspended in lysis buffer (50 mM Tris-HCl pH 7.5, 150 mM NaCl, 5% glycerol, 1 mM EDTA, 2.5 mM MgCl_2_, 1% Triton X-100) supplemented with protease inhibitor cocktail. Crude extracts were obtained by mechanically lysing yeast cells in a fast-prep 3 times for 20 s (6 m/s) with intermediate pauses on ice. Cells were centrifuged at 10,000 x rcf and the supernatant was collected of which the protein concentration was determined. On average, 1 mg protein extract was incubated with 25 µL GST(-Hxk2)-coated beads overnight on a roller drum at 4°C. Finally, beads were washed three times with lysis buffer and the final bead pellet was resuspended in 100 µL 2x loading dye (4% SDS, 100 mM Tris-HCl pH 6.8, 10% glycerol, 0.02% bromophenol blue) and heated at 45°C for 10 min after which the samples were stored at -20°C. For the Hxt7-HA input sample, a fraction of protein extract was immediately added to 2x loading dye, heated at 45°C and stored at -20°C.

(iii) Western blot analysis of yeast proteins

For western blot analysis of yeast proteins, 10 µg of input and 10 µL of pulldown samples were loaded on a 4-12% Bis-Tris NuPage^™^ gel, *in duplo*. Gels were run at a constant voltage of 120-150 V in NuPage^™^ MOPS SDS running buffer. To verify the presence of GST and GST-Hxk2 in the pulldown samples, the first gel was incubated in 60 mg/mL Coomassie Blue Brilliant solution in 10% acetate for 30 min and washed several times with TBS-T buffer (25 mM Tris-HCl pH 8, 150 mM NaCl, 0.05% v/v Tween-20) until gels became clear. The second gel was blotted in NuPage^™^ MOPS SDS blotting buffer containing 20% (v/v) methanol at a constant 300 mA for 1.5 h. After blotting, the nitrocellulose membrane (Hybond-C extra, GE healthcare) was blocked in 5% (w/v) skimmed milk powder dissolved in TBS-T buffer overnight at 4°C. Next, the membrane was immune-labeled with 1:1000 anti-HA (Roche) and washed three times with TBS-T to prepare the membrane for chemiluminescence detection. For this, SuperSignal West Pico PLUS Chemiluminescent substrate (Pierce^®^) was added onto the nitrocellulose membrane and incubated for 2 min in the dark prior to visualization. Chemiluminescence detection was performed using the ImageQuant^™^ LAS4000 mini digital system.

### Determination of hexokinase activity in yeast cell extracts

*In vitro* hexokinase activity of yeast cells was determined as described previously (44, 76). As such, the yeast cells were first grown to exponential phase on Complete Synthetic medium supplemented with 110 mM galactose. The cells were harvested and washed with ice-cold 25 mM MES buffer pH 6. Subsequently, cells were resuspended in lysis buffer (50 mM HEPES pH 7, 150 mM NaCl, 2.5 mM MgCl_2_, 5% glycerol and 1% Triton X-100) containing protease inhibitor after which the yeast cells were mechanically lysed. After protein level determination, total protein concentration was diluted to 0.1 mg/mL in reaction buffer (50 mM HEPES pH 7, 150 mM NaCl, 2.5 mM MgCl_2_, 5% glycerol). For measuring activity, reaction buffer was supplemented with 1.07 mM NADP^+^ and 10 U/mL Glu6P dehydrogenase (Sigma-Aldrich G7877). After temperature equilibration of the plate at 30°C, the reaction was started by adding both ATP and glucose to the reaction buffer. OD_340_ was measured using the Synergy H1 Hybrid reader and hexokinase activity was determined based on the linear increase of absorbance in the first 10-20 s.

### Evaluation of Warbicin^®^ toxicity *in vivo* in mice

Warbicin**^®^** *in vivo* toxicity and tolerability was examined by subjecting female NMRI-nu mice (12 weeks old) to daily intraperitoneal injection of either WBC-A, WBC-15C, WBC-4C or WBC-11C. Compounds were dosed at either 5 mg/kg, 10 mg/kg or 20 mg/kg over a period of 20 days. Three mice were used per condition. To evaluate toxicity, change in body weight was registered daily. As a humane endpoint, mice that lost more than 20% of their original body weight were prematurely sacrificed. In addition, liver function was examined by measuring the ratio of the AST to ALT enzyme activities (AbCam ab105135, ab105134) in sera samples collected at the end of the experiment. Furthermore, the blood glucose level in mice sera was determined using the colorimetric GOD-PAP method. Due to their considerable hydrophobicity, the compounds were dissolved in a sterilized 1xPBS, 5% DMSO and 5% Tween-80 solution until a homogenous suspension was obtained that could be administered in a reproducible way.

## Results

### Reduction of glucose uptake restores growth of the yeast *tps1Δ* strain on glucose

**Figure 1:**
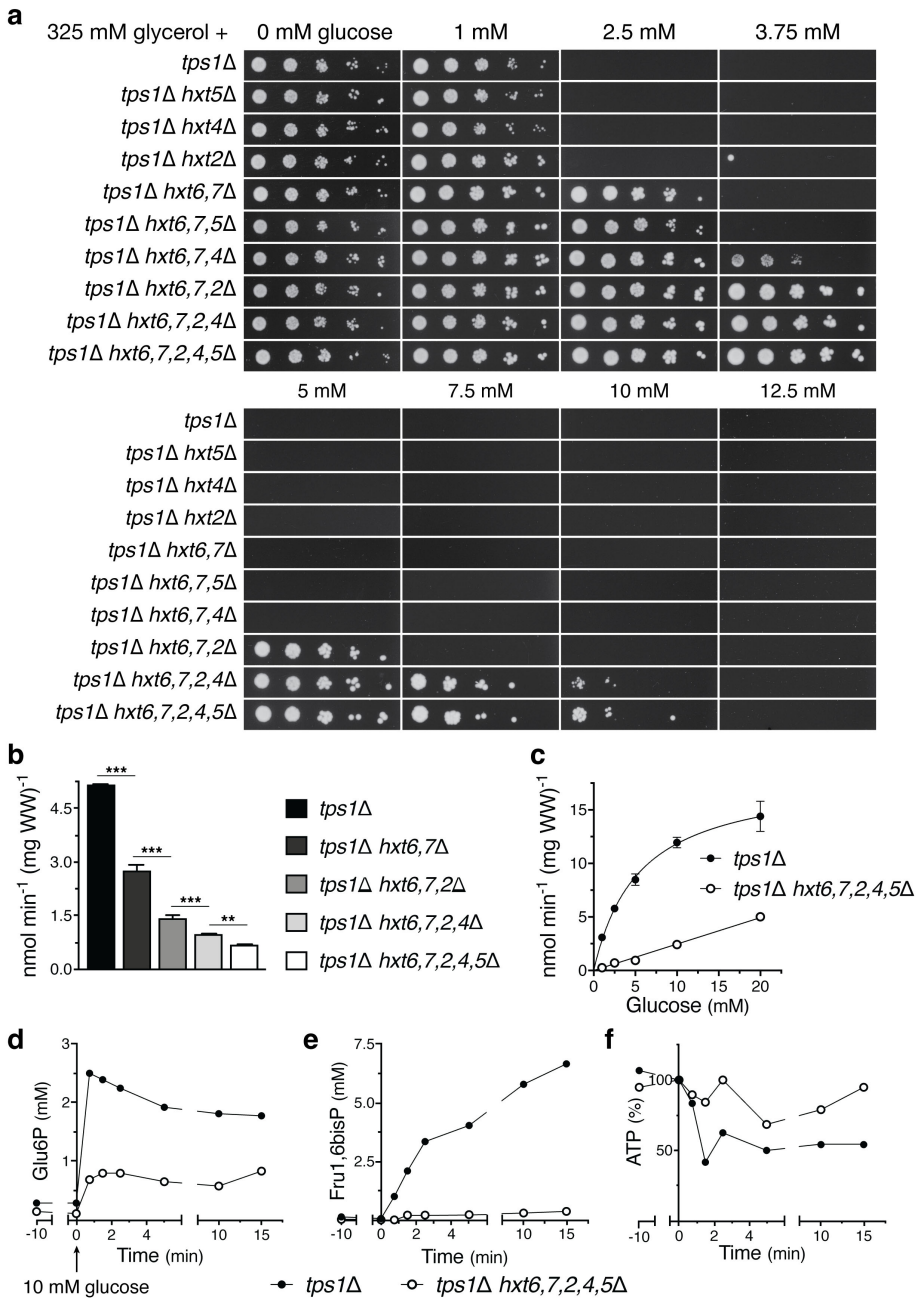
Reducing glucose transport in the yeast *tps1Δ* strain rescues growth on glucose. **(A)** Spot assay displaying the glucose sensitivity of different *hxtΔ* mutants in the yeast *tps1Δ* background. Cells were spotted in five-fold dilutions on plates containing 325 mM glycerol supplemented with the indicated glucose concentrations. **(B)** Uptake of 2.5 mM glucose was measured in yeast *tps1Δ* and *tps1Δ hxtΔ* strains. One-way ANOVA statistical analysis showed a significant decrease in glucose uptake when comparing the effect of additional *HXT* deletion (⁎⁎, *p* <.01; ⁎⁎⁎, *p* <.001). **(C)** Kinetic analysis for glucose uptake of *tps1Δ* and *tps1Δ hxt6,7,2,4,5Δ* cells. **(D–F)** metabolite profiles for **(D)** Glu6P, **(E)** Fru1,6bisP and **(F)** ATP were measured after addition of 10 mM glucose to *tps1Δ* and *tps1Δ hxt6,7,2,4,5Δ* cells. (**C–F)** A representative result of two biological repeats with similar results is shown. For all experiments, cells were (pre)grown on YP medium containing 325 mM glycerol.

We have introduced deletions of *HXT* glucose carrier genes in the yeast *tps1Δ* strain and assessed the effect on its glucose sensitivity. The yeast strains were spotted in serial dilutions on solid nutrient plates containing the nonfermentable carbon source glycerol (325 mM) supplemented with either no glucose or increasing concentrations of glucose, from 1 to 12.5 mM (**Figure 1A**). The results show that individual deletion of the *HXT5*, *HXT4* and *HXT2* genes, encoding intermediate-affinity glucose carriers is unable to restore growth on 2.5 mM glucose. On the other hand, deletion of the *HXT6* and *HXT7* genes, encoding the main high-affinity glucose carriers, and additional deletion of *HXT5*, *HXT4* and/or *HXT2* causes a progressive restoration of growth in the presence of increasing glucose concentrations, with additional deletion of *HXT2* having the strongest effect. These results show that inactivation of glucose carrier genes is able to lower the sensitivity of the yeast *tps1Δ* mutant to glucose. Direct measurements of radioactive glucose uptake in zero *trans*-influx experiments (2.5 mM for 10 s) confirmed that the consecutive deletion of the glucose carrier genes gradually further reduced glucose uptake capacity (**Figure 1B**). The decrease in glucose uptake in the *tps1Δ hxt6, 7, 2, 4, 5Δ* strain compared to the *tps1Δ* strain was most pronounced at the lowest glucose concentrations (**Figure 1C**), explaining the inability of the *tps1Δ hxt6, 7, 2, 4, 5Δ* strain to grow at glucose concentrations higher than 10 mM (**Figure 1A**). The strong decrease in glucose uptake in the *tps1Δ hxt6, 7, 2, 4, 5Δ* strain was correlated with lower levels of Glu6P and Fru1,6bisP, and higher levels of ATP after addition of 10 mM glucose (**Figures 1D-F**). Especially the very strong decrease in Fru1,6bisP was striking. These results show that reduction of glucose uptake can overcome to some extent the growth defect and sugar phosphate overaccumulation of the yeast *tps1Δ* strain on glucose.

### Small-molecule screening for restoration of growth on glucose in the yeast *tps1Δ* strain

**Figure 2:**
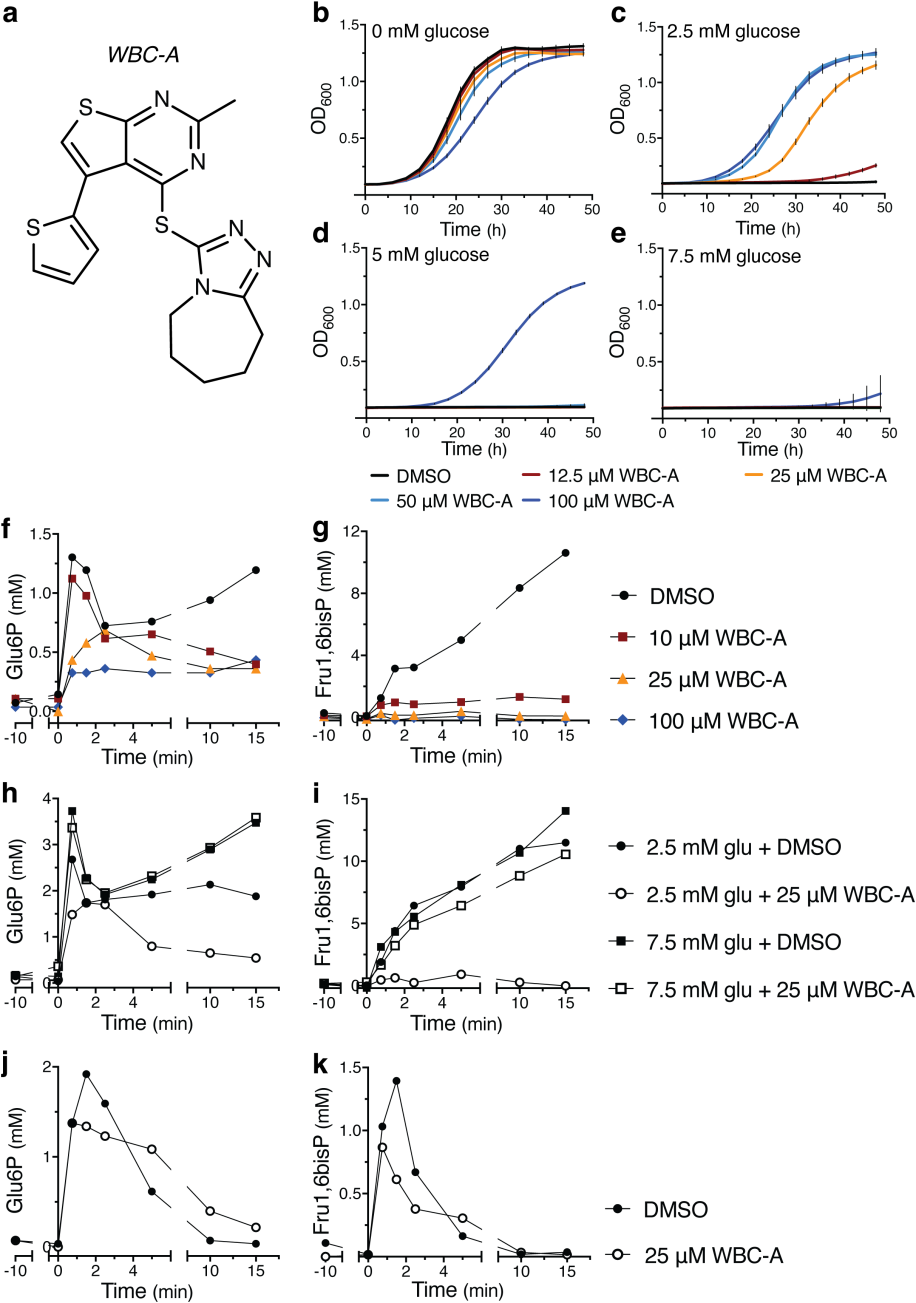
WBC-A restores growth on glucose and lowers glycolytic metabolite accumulation in yeast *tps1Δ* cells. **(A)** Chemical structure of WBC-A. **(B–E)** Growth of yeast *tps1Δ* cells on 110 mM galactose supplemented with different glucose concentrations. Cells were treated with either DMSO (black lines), 12.5 µM WBC-A (red lines), 25 µM WBC-A (orange lines), 50 µM WBC-A (light blue lines) or 100 µM WBC-A (dark blue lines). Metabolic profiles are shown for Glu6P **(F, H, J)** and Fru1,6bisP **(G, I, K)** accumulation as a function of time after addition of glucose at time point zero. Compounds were added at -10 min. **(F, G)** *tps1Δ* cells were given 2.5 mM glucose in the absence or presence of 10 µM, 25 µM or 100 µM WBC-A. **(H, I)** *tps1Δ* cells were treated with DMSO or 25 µM WBC-A after which 2.5 mM glucose or 7.5 mM glucose was added at time zero. **(J, K)** Wild type cells were given 2.5 mM glucose in the absence or presence of 25 µM WBC-A. For all experiments, cells were (pre)grown in Complete Synthetic medium containing 110 mM galactose. Cells were resuspended in the same medium for 30 min prior to glucose addition.

We have screened 40,000 small molecule compounds from a very diverse collection (see Materials and Methods) at the VIB core service screening facility for restoration of growth of the yeast *tps1Δ* strain in the presence of 5 mM glucose in liquid YP medium with 110 mM galactose. Only one single compound with reproducible rescue capacity at the threshold of 5 mM glucose could be isolated and was later called Warbicin**^®^** A (WBC-A). All other initial hits turned out to be false positives or compounds giving irregular rescue of the *tps1Δ* strain. The structure of WBC-A has a part that resembles the structure of adenosine (**Figure 2A**). Addition of WBC-A in the absence of glucose caused insignificant inhibition of yeast growth on galactose, except for the highest concentration of 100 μM, which caused significant growth delay compared to the DMSO control (**Figure 2B**). Only 100 μM WBC-A rescued growth on galactose in the presence of 5 mM glucose while lower concentrations rescued growth up to 2.5 mM glucose. On the other hand, 100 μM WBC-A was not able to restore growth of yeast in the presence of 7.5 mM glucose. Next, we determined the effect of WBC-A on the unusually high accumulation of Glu6P and Fru1,6bisP (several-fold higher than in wild type cells) that is observed after addition of glucose to *tps1Δ* cells. Determination of the level of Glu6P and Fru1,6bisP as a function of time after addition of 2.5 mM glucose to yeast *tps1Δ* cells in medium with 110 mM galactose showed that addition of different concentrations of WBC-A caused a concentration-dependent gradual drop in Glu6P and a more precipitous drop in Fru1,6bisP (**Figures 2F, G**). The drop was dependent on the concentration of glucose. When 7.5 mM glucose was added, the presence of 25 μM WBC-A had little effect on the increase in Glu6P and Frus1,6bisP, as opposed to addition of 2.5 mM glucose (**Figures 2H, I**). This is consistent with the inability of WBC-A to rescue the growth of yeast *tps1Δ* cells in the presence of higher glucose concentrations (**Figures 2B–E**). On the other hand, addition of 25 μM WBC-A to wild type yeast cells caused a much smaller reduction in the increase of Glu6P and Fru1,6bisP after addition of 2.5 mM glucose (**Figures 2J, K**) compared to the effect in *tps1Δ* cells (**Figures 2F, G**). This shows that the overactive glucose phosphorylation activity in yeast *tps1Δ* cells was much more sensitive to WBC-A than the regular glucose phosphorylation activity in wild type yeast cells. Our results therefore show that we have been able to isolate a compound that restores growth on glucose of the yeast *tps1Δ* strain by counteracting its overactive glucose influx into glycolysis and that the compound therefore might more preferentially target overactive glucose catabolism compared to basal glucose catabolism.

### Warbicin^®^ A acts through inhibition of glucose uptake

**Figure 3:**
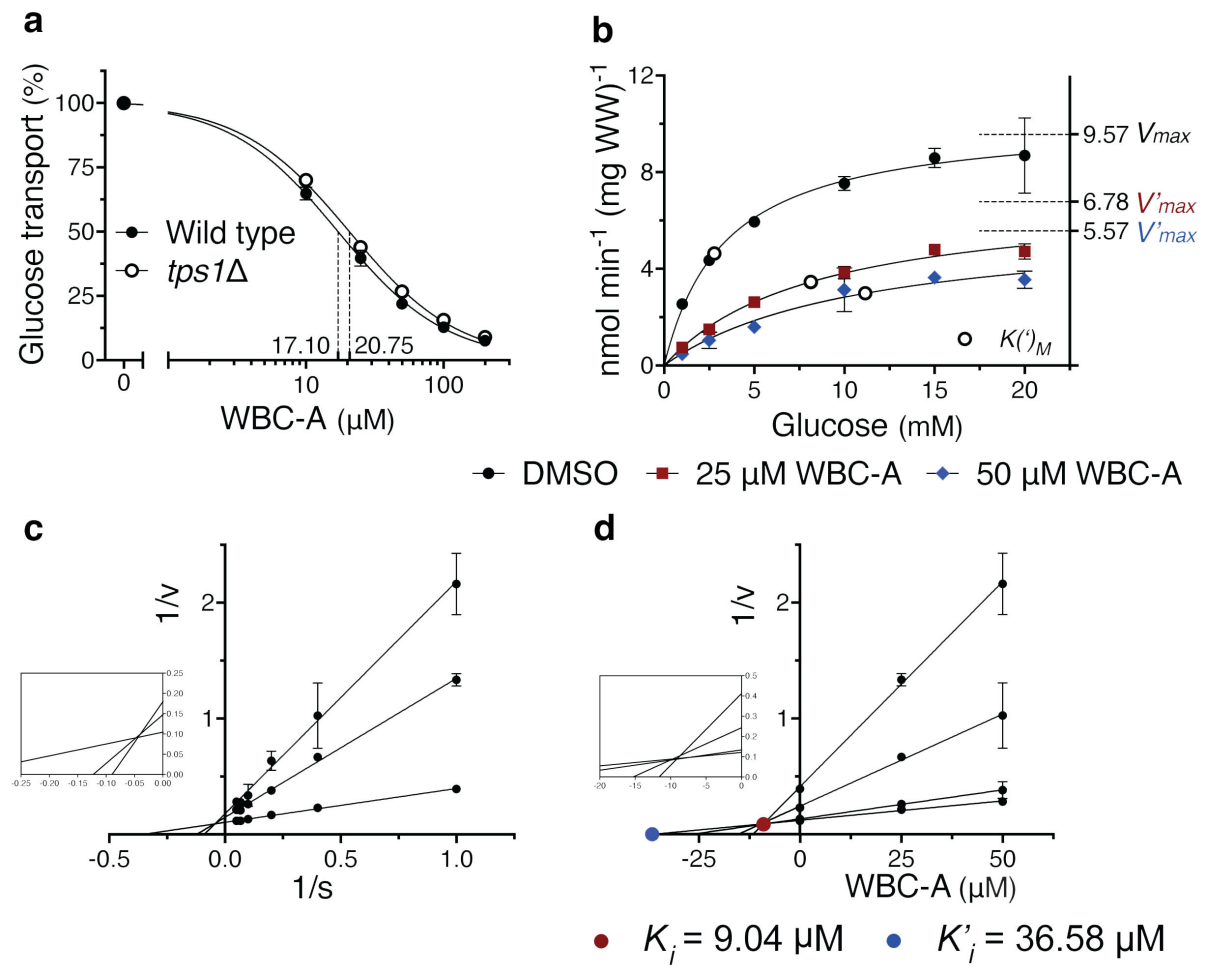
WBC-A inhibits glucose uptake of wild type and *tps1Δ* yeast cells with mixed type inhibition. **(A)** Dose-response inhibition of 2.5 mM glucose uptake by WBC-A in wild type and *tps1Δ* yeast cells. IC_50_ values are indicated by dashed lines. **(B)** Kinetic analysis of glucose uptake in wild type cells in the absence or presence of 25 µM or 50 µM WBC-A. Corresponding *V(‘)max* (dashed lines) and *K(‘)_M_* (open circles) values are indicated. **(C)** Lineweaver-Burk plot analysis using the data of **(B)** for wild type yeast cells for the determination of mode-of-inhibition. **(D)** Corresponding Dixon plot analysis for estimation of the *K_i_* (closed red circle) and *K’_i_* (closed blue circle) inhibitor constants of WBC-A. For all experiments, cells were (pre)grown on Complete Synthetic medium containing 110 mM galactose.

The inhibition of sugar phosphate hyperaccumulation by WBC-A in yeast *tps1Δ* cells suggested that WBC-A may act by reducing glucose uptake. We have measured the effect of different concentrations of WBC-A on uptake activity of 2.5 mM glucose by wild type and *tps1Δ* yeast cells using radioactive glucose with a 10 s time scale. WBC-A reduced glucose uptake activity similarly in wild type and *tps1Δ* cells with an IC_50_ of 17.10 μM and 20.75 μM, respectively (**Figure 3A**). Initial glucose uptake activity also did not differ significantly. Subsequent analysis revealed a mixed type of inhibition by WBC-A. A kinetic analysis was performed of the inhibition of glucose uptake by 25 and 50 µM WBC-A using different glucose concentrations (**Figure 3B**). Lineweaver-Burk plot analysis revealed a mixed type of inhibition (**Figure 3C**) and using Dixon plot analysis we determined a *K_i_* of 9.04 μM and a *K’_i_* of 36.58 μM (**Figure 3D**). The same analysis was performed for glucose uptake in yeast *tps1Δ* cells, which also revealed a mixed type of inhibition with very similar inhibitor constants of 8.35 μM for *K_i_* and 35.44 μM for *K’_i_* (**Supplementary Figure 1**). This showed that inhibition of glucose uptake by WBC-A is independent of Tps1.

To evaluate whether WBC-A may also act through inhibition of hexokinase activity in yeast, we determined the effect of WBC-A on hexokinase activity measured in extracts of wild type yeast cells (**Supplementary Figure 2**). A variable glucose concentration and fixed ATP concentration, as well as a variable ATP concentration and fixed glucose concentration were used. No significant effect of WBC-A on *in vitro* hexokinase activity of yeast could be detected, further supporting the conclusion that WBC-A acts through inhibition of glucose uptake. Hence, we can conclude that WBC-A is an inhibitor of glucose uptake, likely acting directly on the glucose carriers and not by inhibiting the downstream glucose phosphorylating enzymes.

### Warbicin^®^ A analogs rescue growth on glucose of yeast *tps1Δ* cells and inhibit glucose uptake in yeast with varying potency

**Figure 4:**
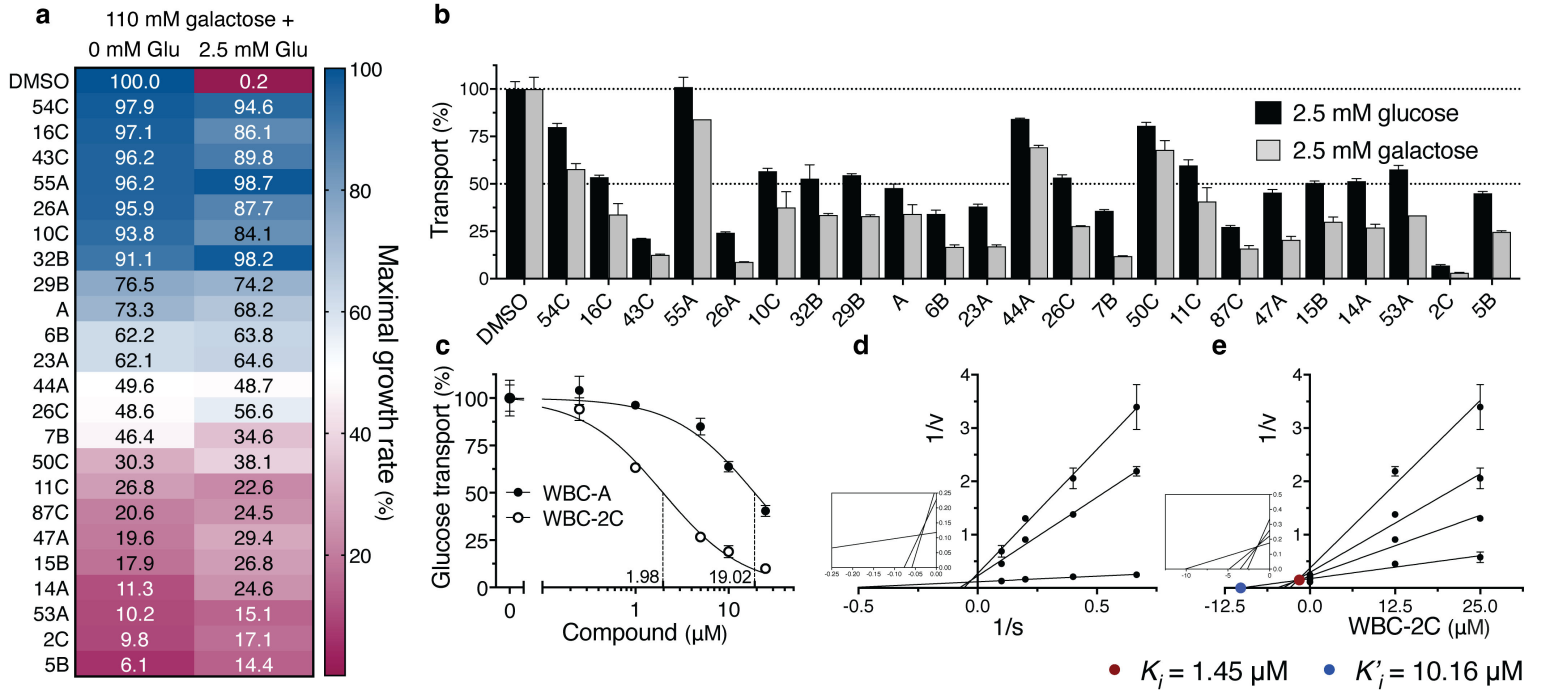
Structural analogs of WBC-A rescue growth and inhibit glucose uptake of yeast *tps1Δ* cells to varying degrees. **(A)** Structural analogs of WBC-A that rescued growth of the yeast *tps1Δ* strain were compared for their influence on maximal growth rate relative to the control (DMSO). Compounds were added at 100 µM to *tps1Δ* cells growing in medium containing 110 mM galactose or 110 mM galactose supplemented with 2.5 mM glucose. **(B)** Inhibition of 2.5 mM glucose or 2.5 mM galactose uptake in yeast *tps1Δ* cells by 25 µM of various WBC compounds. **(C)** Dose-response inhibition of 2.5 mM glucose transport in wild type yeast cells by WBC-A and WBC-2C. IC_50_s are indicated by dashed lines. **(D)** Lineweaver-Burk plot analysis for determination of mode-of-inhibition by WBC-2C for wild type cells. **(E)** Corresponding Dixon plot analysis for estimation of the *K_i_* (closed red circle) and *K’_i_* (closed blue circle) inhibitor constants of WBC-2C. For all experiments, cells were (pre)grown on Complete Synthetic medium containing 110 mM galactose.

Next, we screened a collection of about 200 structural analogs of WBC-A in a concentration of 100 μM for the capacity to restore growth of the yeast *tps1Δ* mutant in 110 mM galactose liquid medium containing 2.5 mM glucose (**Figure 4A**). Growth on 110 mM galactose in the presence of DMSO was used as reference (100%) and addition of 2.5 mM glucose in the presence of only DMSO virtually eliminated growth. Several analogs showed higher potency in this assay compared to WBC-A and they also caused less inhibition of yeast growth on 110 mM galactose in the absence of 2.5 mM glucose (**Figure 4A**). The reverse was seen with a series of other analogs. They showed less potency in restoring growth of the yeast *tps1Δ* mutant in the presence of 2.5 mM glucose than WBC-A and reduced growth on 110 mM galactose in the absence of glucose more than WBC-A (**Figure 4A**). It should be noted that galactose is taken up in yeast by the Gal2 galactose/glucose carrier, which is most closely related to the high-affinity Hxt6 and Hxt7 glucose carriers among the members of the Hxt glucose carrier family (25, 77). Hence, cross-inhibition of WBCs against Hxt glucose carriers and Gal2 can certainly not be excluded. Indeed, uptake of 2.5 mM glucose or 2.5 mM galactose in *tps1Δ* cells was inhibited in a very similar way by a series of WBCs at a concentration of 25 µM in zero *trans*-influx experiments (**Figure 4B**). Inhibition of galactose uptake was always more pronounced compared to glucose uptake, possibly because there is only a single galactose carrier, Gal2, versus many Hxt glucose carriers. Kinetic analysis of the inhibition of 2.5 mM glucose uptake by wild type cells showed that WBC-2C was a much more potent inhibitor with an IC_50_ of 1.98 µM versus 19.02 µM for WBC-A (**Figure 4C**). Lineweaver-Burk plot analysis revealed a mixed type of inhibition also for WBC-2C (**Figure 4D**). Dixon plot analysis showed a *K_i_* value of 1.45 µM and a *K’_i_* of 10.16 µM for inhibition by WBC-2C (**Figure 4E**).

We also evaluated for general toxicity of the WBC compounds in yeast. For that purpose, we tested the effect of a relatively high concentration of 100 µM on growth of wild type yeast cells on a fermentative medium with glucose (110 mM) and on a respirative medium with 325 mM glycerol + 430 mM ethanol (**Supplementary Figure 3**). The reference compound WBC-A did not cause any growth inhibition at all on the two media. In fact, none of the compounds caused more than 50% reduction in growth. Inhibition on glucose medium was always somewhat more pronounced compared to that on respirative medium, with 55A, 23A and 7B as the most conspicuous exceptions. The majority of the Warbicin**^®^** compounds even caused an apparent slight stimulation of growth compared to the DMSO control (**Supplementary Figure 3**). The structure of WBC-A and the 21 analogs that reduce glucose uptake is shown in **Supplementary Table 1**, with their corresponding IC_50_s for 2.5 mM glucose transport inhibition and the minimal rescue concentration for growth of the *tps1Δ* strain on 2.5 mM glucose. Strikingly, these 21 compounds share a common backbone structure with WBC-A, that is likely important for bioactivity. WBC-55A was the only compound with a variation in the backbone structure (**Supplementary Figure 4A**). Although it caused the best rescue of *tps1Δ* cells on 2.5 mM glucose (**Figure 4A**), it did not cause any inhibition of glucose transport in short-term 10 s glucose uptake (**Figure 4B**), which might relate to its deviating backbone structure. In addition, WBC-55A caused only a minimal decrease in the hyperaccumulation of Glu6P and Fru1,6bisP after addition of 2.5 mM glucose, as opposed to the much stronger effect of WBC-A (**Supplementary Figures 4B, C**). This suggests that a downstream target in the signaling pathway, leading from the glycolytic deregulation to growth arrest and apoptosis in the *tps1Δ* mutant (24), is the main target of WBC-55A rather than the glucose uptake system.

These results show that many analogs of WBC-A can be isolated with similar inhibitory potency on glucose uptake in yeast, providing a range of tools for development of new candidate anti-cancer drugs.

### *In vitro* and *in vivo* evidence for physical interaction between Hxt7 and Hxk2 in yeast cells and nuclear localization of Hxk2 rescues growth on low glucose of yeast *tps1Δ* cells

**Figure 5:**
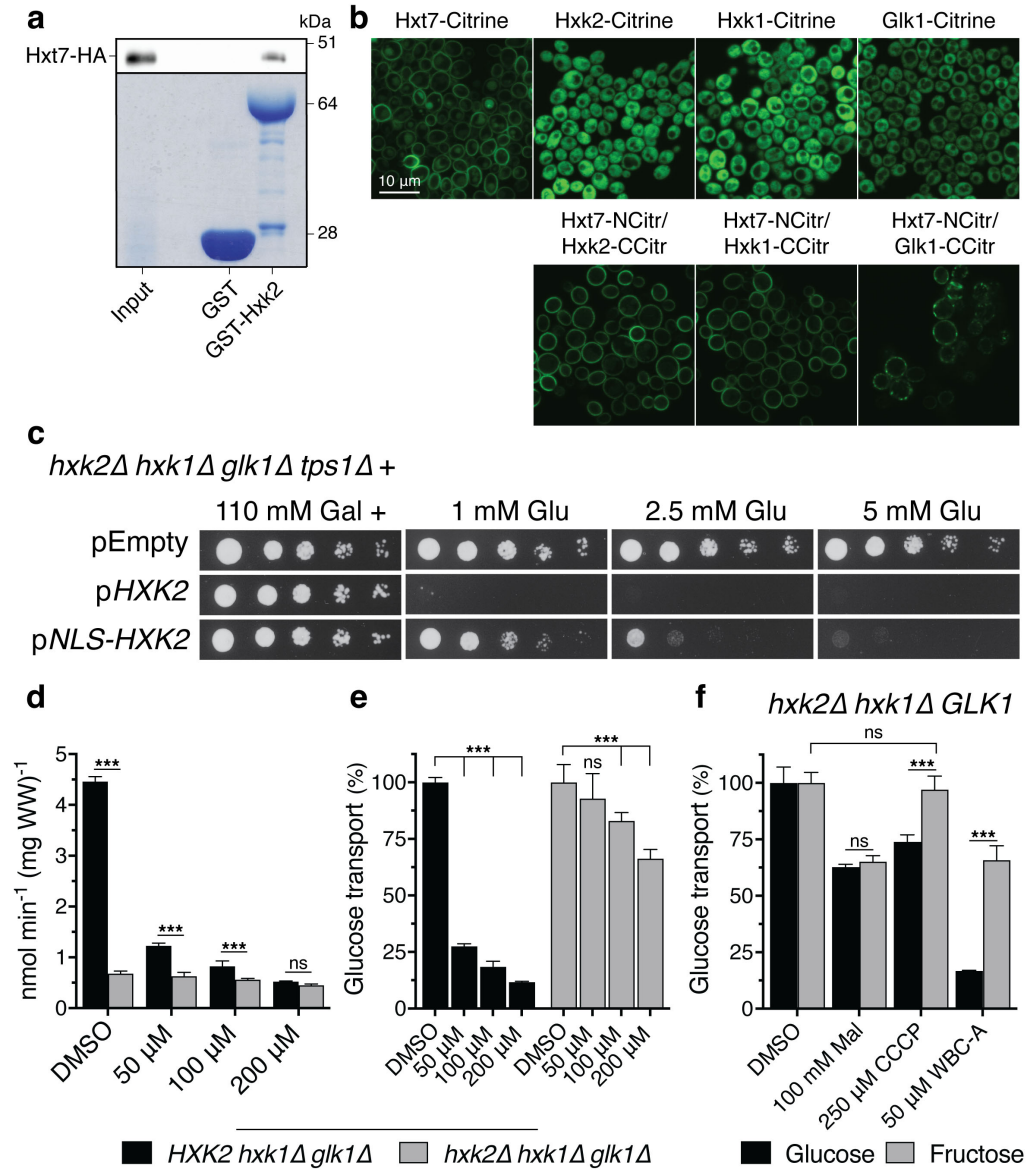
Physical evidence for possible interaction between glucose transport and phosphorylation and its connection with inhibition by WBC-A in yeast cells. **(A)** Pulldown of Hxt7-HA from yeast wild type cell extracts by GST-Hxk2. Presence of Hxt7-HA was visualized by western blotting (upper panel), whereas presence of GST and GST-Hxk2 was confirmed by Coomassie blue staining (lower panel). **(B)** Fluorescence microscopy images of BiFC interactions between Hxt7 and Hxk2, Hxk1 or Glk1 at the level of the yeast cell plasma membrane as well as the cytosolic localization of the three sugar kinases fused to full-length Citrine. **(C)** Spot assay displaying the glucose sensitivity of *hxk2Δ hxk1Δ glk1Δ tps1Δ* yeast cells transformed with a vector with either no insert, a *HXK2* allele or a *NLS-HXK2* allele. All plates contained 110 mM galactose and were supplemented with the indicated glucose concentration. Pictures were taken after three days. **(D)** Uptake inhibition of 1 mM glucose by DMSO or different concentrations of WBC-A was measured in *HXK2 hxk1Δ glk1Δ* and *hxk2Δ hxk1Δ glk1Δ* yeast cells. **(E)** Uptake rates in **(D)** are put relative to DMSO treated yeast cells (100%) for each strain. **(F)** Uptake rates of glucose and fructose at tracer concentration were measured in *hxk2Δ hxk1Δ GLK1* yeast cells. DMSO, 250 µM CCCP and 50 µM WBC-A were given 10 min prior to the uptake measurement except for 100 mM maltose which was given simultaneously with the tracer sugar. Significance was determined by two-way ANOVA followed by Sidak’s multiple comparisons test (***, *p* <.001; ns, non-significant). For all experiments, the yeast cells were (pre)grown on medium with 325 mM glycerol and 430 mM ethanol except for the spot assay where cells were (pre)grown on 110 mM galactose in uracil-deficient medium for plasmid retention.

Our next aim was to investigate whether there might still be a connection between the inhibitory effect of the Warbicin^®^ compounds and glucose phosphorylation, in spite of the fact that WBC-A had no effect on hexokinase activity in yeast cell extracts (**Supplementary Figure 2**). Hence, we have investigated possible interaction between the Hxt7 glucose carrier and the hexokinase Hxk2 in yeast cells (**Figure 5**). Using a pulldown experiment with GST-labeled Hxk2, we could precipitate HA-labeled Hxt7, indicating *in vitro* physical interaction between the two proteins (**Figure 5A**). Moreover, Hxt7 interacts *in vivo* with the three sugar kinases, Hxk2, Hxk1 and Glk1, as shown by Bimolecular Fluorescence Complementation (BiFC) (**Figure 5B**). This technology is based on the emission of fluorescence when the two separated, non-fluorescent halves of Citrin fused to two candidate interacting proteins are brought together by physical binding of the two interacting proteins. Control experiments with confocal microscopy show that the Hxk1-, Hxk2- and Glk1-Citrine fusion proteins are located in the cytosol of the yeast cells, while the Hxt7-Citrine fusion protein is located at the level of the plasma membrane (**Figure 5B**). On the other hand, yeast cells expressing the fusion proteins of the N-terminal part of Citrine with Hxt7 and fusion proteins of the C-terminal part of Citrine with either Hxk1, Hxk2 or Glk1, all display fluorescence at the level of the plasma membrane (**Figure 5B**). Additional control experiments confirmed that Hxt7 fused to the N-terminal part of Citrine does not spontaneously assemble with the C-terminal part of Citrine expressed freely in the cytosol (**Supplementary Figure 5**). The BIFC results support that the yeast sugar kinases physically interact *in vivo* with the yeast Hxt7 glucose transporter. To evaluate whether physical interaction of Hxk2 with Hxt7 may play a role in the growth defect of the yeast *tps1Δ strain* on glucose, we fused a Nuclear Localization Sequence (NLS) sequence to Hxk2 and expressed the fusion protein in a *hxk1Δ hxk2Δ glk1Δ* strain. Whereas the Hxk2-Citrine protein was only present in the cytosol, the NLS-Hxk2-Citrine protein was confined to the nucleus (**Supplementary Figure 6A**). The fusion of the SV40 large T-antigen NLS sequence (78) to Hxk2 and the nuclear localization of NLS-Hxk2 did not affect growth of the yeast, neither on low nor on high glucose concentrations, indicating that hexokinase activity *in vivo* was not compromised (**Supplementary Figure 6B**). We next expressed Hxk2 and NLS-Hxk2 in the yeast *hxk1Δ hxk2Δ glk1Δ tps1Δ* strain. This showed that confinement of NLS-Hxk2 to the nucleus rescued to some extent the growth defect of the yeast *tps1Δ* strain on low glucose concentrations (**Figure 5C**). This suggests that physical interaction between hexokinase and the Hxt7 glucose carrier is at least to some extent involved in the growth defect of the yeast *tps1Δ* strain on glucose. Although NLS addition clearly caused sorting of Hxk2 to the nucleus, some Hxk2 may remain in the cytosol (in addition to Hxk1), which could explain why rescue of the *tps1Δ* strain is limited to the lower glucose concentrations.

These results show that the Hxt7 glucose carrier physically interacts with the glucose phosphorylating enzymes in yeast and that direct glucose carrier hexokinase interaction appears to be important for the glucose growth defect of the yeast *tps1Δ* mutant.

### Inhibition of glucose uptake by WBC-A in yeast cells is dependent on glucose phosphorylation

Our next aim was to investigate the possible connection with glucose phosphorylation further. Hence, we investigated whether the inhibition of glucose uptake by WBC-A in yeast cells might be dependent on hexokinase activity. The glucose uptake rate in cells of the *hxk1Δ hxk2Δ glk1Δ* yeast strain was reduced compared to that of the *HXK2 hxk1Δ glk1Δ* strain (**Figure 5D**), consistent with previous literature data (79). Addition of WBC-A caused little further inhibition in the strain lacking glucose phosphorylation, while in the yeast strain expressing Hxk2 and thus possessing active glucose phosphorylation, a strong decrease in glucose uptake rate was observed (**Figures 5D, E**). These results seemed to suggest that in the absence of hexokinase activity, the carrier is much less inhibited by WBC-A and that WBC-A therefore might target an additional process rather than just the activity of the glucose carrier. To address this in more detail, uptake of glucose and fructose was measured in *hxk2Δ hxk1Δ GLK1* yeast cells, which have active glucose phosphorylation but no active fructose phosphorylation (**Figure 5F**). Here, hexose uptake was measured at tracer concentration to minimize the backflow of hexose sugar that sets in when no functional hexokinase activity is present. A decrease of the cellular ATP level caused by addition of 250 µM of the protonophore CCCP lowered the glucose uptake rate but not that of fructose, which is in line with fructose not being phosphorylated by Glk1. The addition of maltose, a competitive inhibitor of glucose and fructose uptake, caused a similar decrease in the uptake of the two sugars (**Figure 5F**). WBC-A caused strong inhibition of glucose uptake but little inhibition of fructose uptake in this yeast strain (**Figure 5F**). On the other hand, fructose transport was much more inhibited by WBC-A in a yeast strain only expressing *HXK2* (> 75%) compared to a strain expressing only *GLK1* (± 30%) (**Supplementary Figures 7A, B**). Although glucose is the main substrate of glucokinase, it may have some residual fructose phosphorylating activity. Also, inhibition by WBC-A of galactose uptake, which is mediated in yeast by the Gal2 galactose/glucose permease, a member of the Hxt family, was to some extent dependent on galactose phosphorylation. The latter is mainly mediated by the galactokinase Gal1 (25). Deletion of the regulatory genes Gal80 and/or Gal3 of the Gal system, caused little reduction of WBC-A inhibitory potency (**Supplementary Figures 7C, D**).

These results reveal the importance of sugar phosphorylation for inhibition of sugar uptake by WBC-A in yeast cells and they support the concept that WBC-A inhibits glucose uptake by targeting glucose carrier hexokinase interaction, as for instance happens in transport-associated phosphorylation of sugar.

### Warbicin^®^ A inhibits the human GLUT1 and GLUT4 glucose transporters expressed in a yeast glucose-transporter null strain

**Figure 6:**
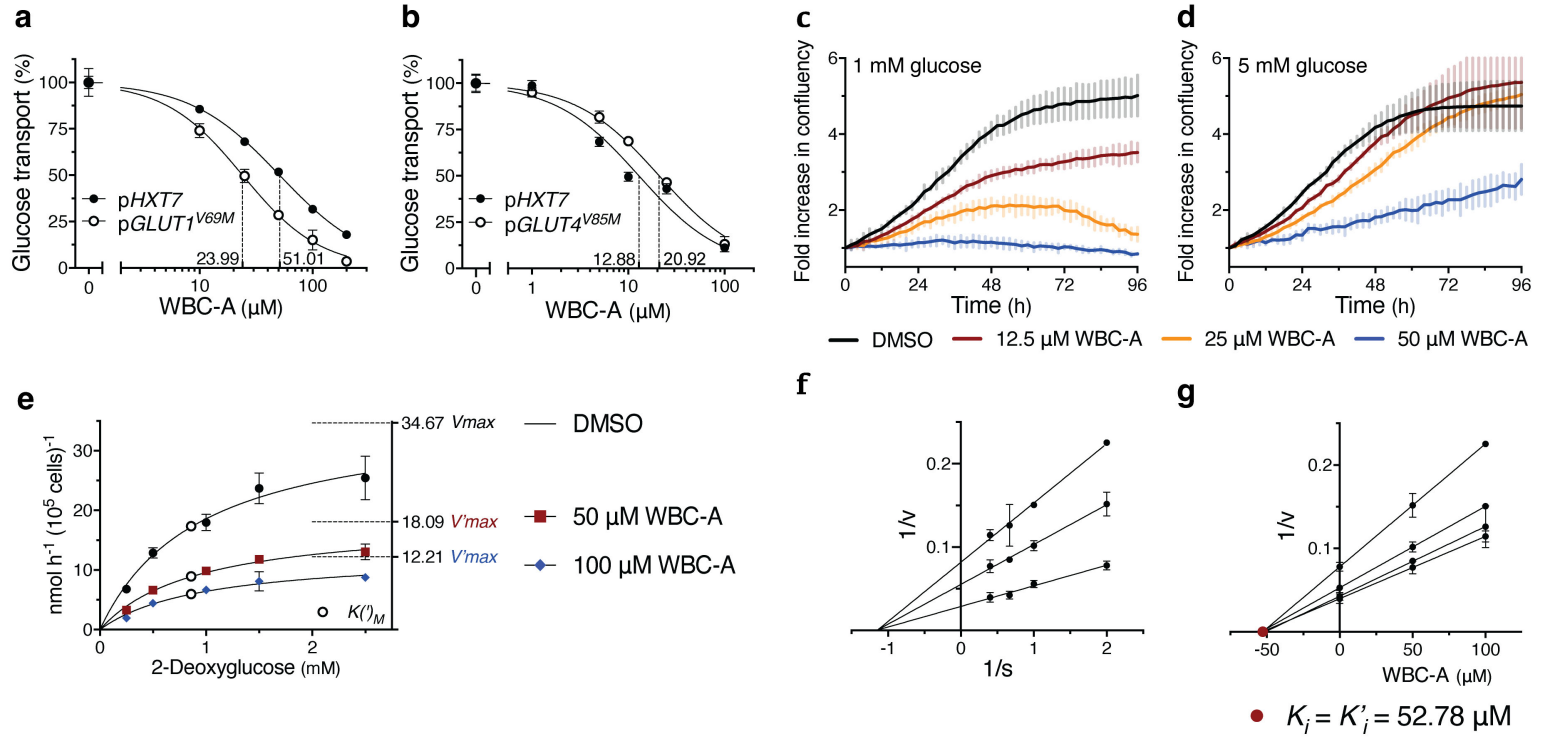
WBC-A inhibits GLUT activity and growth and glucose uptake of A549 lung adenocarcinoma cells. **(A)** Dose-response inhibition of 2.5 mM glucose transport by WBC-A in yeast RE700A *hxt^0^ gal2Δ* cells expressing either p*HXT7* or p*GLUT1^V69M^*. **(B)** Dose-response inhibition of 2.5 mM glucose transport by WBC-A in yeast EBY.VW4000 *hxt^0^ erg4*Δ cells expressing either p*HXT7* or p*GLUT4^V85M^*. IC_50_s **(A, B)** are indicated by dashed lines. **(C, D)** Growth analysis of A549 lung adenocarcinoma cells cultured in RPMI containing either **(C)** 1 mM glucose or **(D)** 5 mM glucose. Cells were treated with 0.1% DMSO, 12.5 µM WBC-A, 25 µM WBC-A or 50 µM WBC-A. Growth is expressed as fold increase in measured confluency. **(E)** Kinetic analysis of 2-deoxyglucose uptake in A549 lung adenocarcinoma cells in the absence or presence of 50 µM WBC-A or 100 µM WBC-A. Corresponding *V(‘)max* (dashed lines) and *K(‘)_M_* (open circles) values are indicated. **(F)** Lineweaver-Burk plot analysis using the data of **(E)** for determination of mode-of-inhibition. **(G)** Corresponding Dixon plot analysis for estimation of the *K_i_* = *K’_i_* (closed red circle) inhibitor constants of WBC-A. To measure glucose uptake by yeast cells expressing GLUT isoforms, cells were (pre)grown in YP medium with 58 mM maltose. A549 cells were precultured in RPMI medium supplemented with 10 mM glucose.

To assess whether WBC-A could inhibit glucose transport activity of the GLUT carriers, we expressed human GLUT1^V69M^ and GLUT4^V85M^ in a yeast *hxt^0^ gal2Δ* (RE700A) and *hxt^0^ erg4Δ* (EBY.VW4000) strain, respectively. Both these yeast strains are completely deficient in glucose uptake due to the absence of all active glucose transporters. For functional GLUT expression in yeast, specific mutations had to be introduced in both sequences, whereas for GLUT4, an additional *ERG4* gene deletion was required (75, 80, 81). WBC-A inhibited glucose uptake of GLUT1^V69M^ expressed in yeast with an IC_50_ of 23.99 µM versus 51.01 µM for the yeast Hxt7 carrier solely expressed in the same *hxt^0^* strain (**Figure 6A**). GLUT4^V85M^ expressed in the other yeast *hxt^0^* genetic background was inhibited by WBC-A with an IC_50_ of 20.92 µM versus 12.88 µM for yeast Hxt7 expressed in the same genetic background (**Figure 6B**). These results show that the mammalian glucose transporters of the GLUT family are also inhibited by WBC-A, suggesting that we have isolated a new drug with a broad action spectrum on eukaryotic glucose transporters of the MFS family.

### WBC-A is the first drug that inhibits glucose uptake both in mammalian and in yeast cells

Finally, we have compared the effect of WCB-A on glucose uptake by yeast Hxt7 and human GLUT1^V69M^ with that of known mammalian glucose uptake inhibitors. For that purpose, Hxt7 and GLUT1^V69M^ were expressed in the *hxt^0^ gal2Δ* strain. All compounds were added at the same concentration of 25 µM. The results showed that the mammalian glucose uptake inhibitors only inhibited GLUT1^V69M^ and not Hxt7, while WBC-A was the only compound that inhibited glucose uptake both by Hxt7 and GLUT1^V69M^ (**Supplementary Figure 8**).

These results suggest that Warbicin^®^ compounds inhibit glucose uptake through a different action mechanism compared to the previously known classical mammalian glucose transport inhibitors and that this mechanism is conserved between yeast Hxt and mammalian GLUT glucose carriers.

### Warbicin^®^ A inhibits proliferation and glucose uptake of cancer cells

To assess whether the inhibitory effect of WBC-A on the mammalian glucose transporters expressed in yeast, was relevant for glucose uptake in mammalian cells, we first tested the effect of WBC-A on the proliferation of A549 lung adenocarcinoma cells. This cell type was chosen based on the previously reported inhibition of its proliferation by the WZB-117 inhibitor of GLUT1 (67). The test was performed in RPMI medium with either 1 mM glucose (**Figure 6C**) or 5 mM glucose (**Figure 6D**). In both cases, WBC-A caused a dose-dependent inhibition of growth, which was more pronounced for 1 mM compared to 5 mM glucose medium. In 1 mM glucose medium, 12.5 µM WBC-A caused pronounced inhibition, while higher concentrations of WBC-A completely blocked continued cancer cell proliferation (**Figure 6C**). Subsequent kinetic analysis of WBC-A inhibition of 2-deoxyglucose uptake in A549 lung adenocarcinoma cells revealed a noncompetitive type of inhibition. Uptake of different 2-deoxyglucose concentrations was inhibited by 50 µM and 100 µM WBC-A in a dose-dependent manner (**Figure 6E**). Lineweaver-Burk plot analysis revealed a noncompetitive type of inhibition (**Figure 6F**) and using Dixon plot analysis, we determined a *K_i_* (= *K’_i_*) of 52.78 μM (**Figure 6G**). These results show that the inhibition by WBC-A of the human GLUT transporters expressed in yeast, also applies to glucose uptake and its requirement for proliferation in human cells.

### Screening of structural analogs of WBC-A for inhibition of cancer cell proliferation

The next aim was to identify structural analogs of WBC-A with stronger inhibitory potency on cancer cell proliferation. For that purpose, we screened 203 structural analogs of WBC-A for inhibition of proliferation on 1 mM glucose of the commonly used A549 lung adenocarcinoma cell line (**Supplementary Figure 9**). DMSO (green bar) and WBC-A (orange bar) served as negative and positive control, respectively. We also included in this assay five known inhibitors of mammalian glucose uptake: STF-31, Fasentin, BAY-876, WZB-117 and Cytochalasin B. All compounds were tested in a concentration of 50 µM. A large variation in effectiveness for growth inhibition was observed ranging from apparent stimulation of cell proliferation to stronger inhibition than WBC-A and even Cytochalasin B, the strongest inhibitor of cell proliferation in this assay. We selected the 77 most potent compounds, encompassing the range covered by the known glucose uptake inhibitors, for further analysis. These results show that functional analogs of WBC-A can be selected based on their chemical structure and direct screening for growth inhibition of cancer cells.

### Characterization of structural analogs of WBC-A for inhibition of cancer cell proliferation

**Figure 7:**
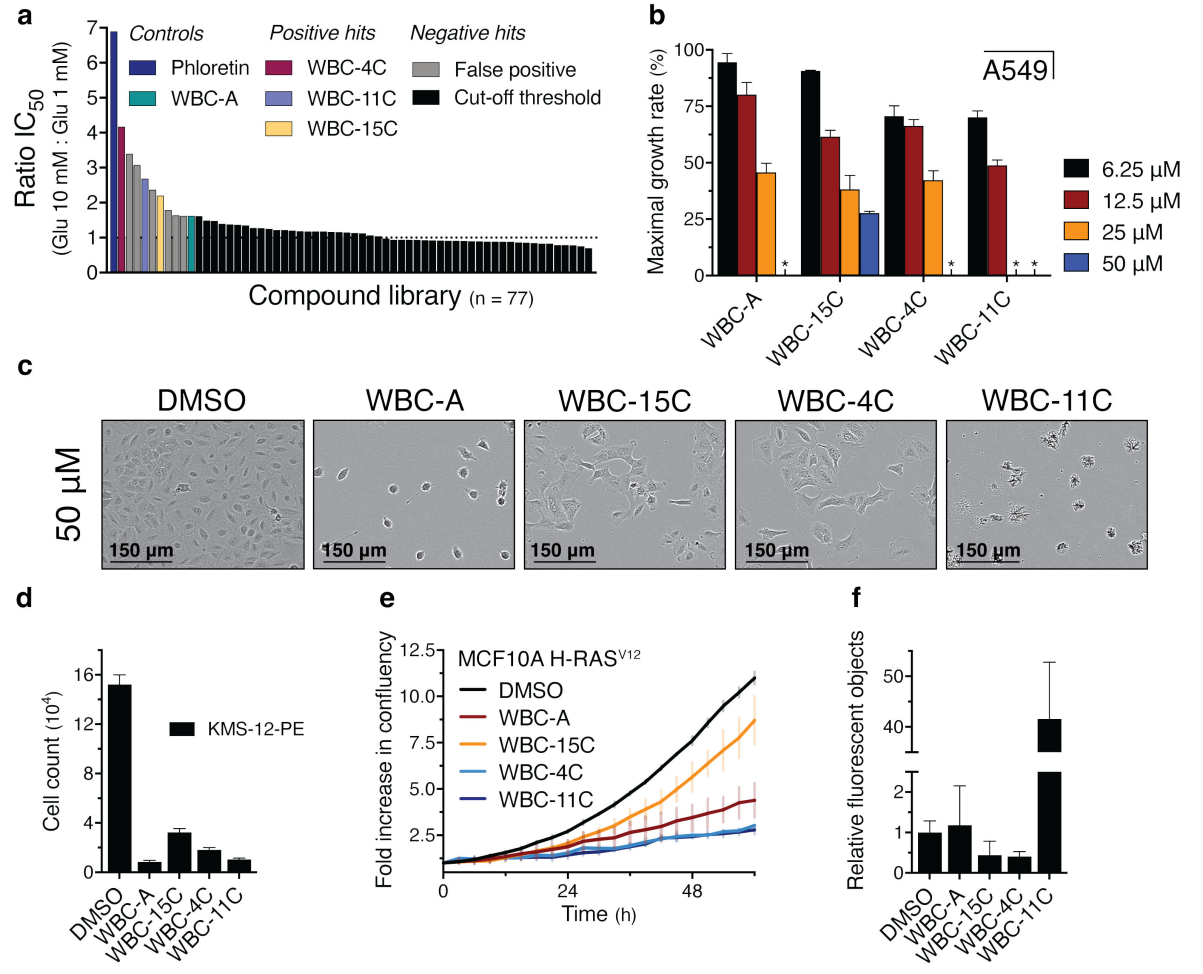
Structural analogs of WBC-A inhibit cell proliferation of different human cancer cell lines. **(A)** The ratio of IC_50_ values for inhibition of A549 lung adenocarcinoma cell proliferation on 10 mM and 1 mM glucose is illustrated for every analog that was isolated from the pre-screening (n = 77, **Supplementary Figure 5**). Phloretin (dark blue bar) was included as a positive control whereas WBC-A (green bar) served as cut-off threshold for selection of compounds with similar or higher potency. **(B)** Maximal growth rates on 1 mM glucose are shown for A549 cells at 6.25 µM, 12.5 µM, 25 µM or 50 µM of either WBC-A, -15C, -4C or -11C. Absence of growth is indicated by an asterisk symbol. Growth was set relative to growth on DMSO (100%). **(C)** Phase contrast microscopy images of A549 cells treated with either DMSO or 50 µM of WBC-A, -15C, -4C or -11C. Pictures were taken after three days. **(D)** Cell count of KMS-12-PE multiple myeloma cells after 4 days of growth on RPMI medium with 1 mM glucose. Cells were treated with either DMSO or 25 µM of WBC-A, -15C, -4C or -11C. **(E)** Growth curve of the MCF10A breast epithelial cell line transformed with the *H-RAS^V12^* allele on 1 mM glucose. Cells were treated with either DMSO, or 25 µM of WBC-A, WBC-15C, WBC-4C or WBC-11C. Growth was based on the increase in confluency as determined by the Incucyte software. **(F)** Relative fluorescent object counts originating from apoptosis induction in MCF10A H-RAS^V12^ cells growing on 1 mM glucose, as determined by the Incucyte software. Cells were treated with either DMSO or 25 µM of WBC-A, -15C, -4C or -11C. Fluorescent object counts after three days were corrected for total confluency percentage and normalized to the DMSO control.

We next determined the ratio of the IC_50_ values for inhibition of A549 lung adenocarcinoma cell proliferation on 10 mM and 1 mM glucose for these analogs identified in the pre-screening (**Figure 7A**). The compounds WBC-4C, WBC-11C and WBC-15C were singled out for further analysis since they were the only compounds among the most potent inhibitors that displayed a reproducible effect upon repetition. The other compounds turned out to be false positives that could not be validated afterwards.

The maximal growth rate of A549 lung adenocarcinoma cells was reduced in a concentration-dependent manner by the Warbicin**^®^** compounds with WBC-11C and WBC-15C being most and least potent, respectively, while WBC-A and WBC-4C had similar, intermediate potency (**Figure 7B**).

Microscopy pictures of A549 cells revealed aberrant cell morphology to different extents in the presence of the highest concentration of 50 µM of the compounds (**Figure 7C**). It is not clear whether this is due to very stringent inhibition of glucose uptake or to toxic side-effects of the compounds at high concentrations.

These results show that we were able to select several functional analogs of WBC-A with a reliable inhibitory potency on cancer cell proliferation.

### Inhibition of proliferation in other cancer cell types

We next assessed whether the Warbicin^®^ compounds could have a general inhibitory effect on different cancer types. Proliferation of the multiple myeloma KMS-12-PE cell line on 1 mM glucose was also severely inhibited by 25 µM of the four Warbicin**^®^** compounds (**Figure 7D**). Proliferation of an MCF10A breast epithelial cell line transformed with the *H-RAS^V12^* allele (71) on 1 mM glucose was inhibited by the four compounds with WBC-4C and WBC-11C showing the highest potency (**Figure 7E**). In the same medium, WBC-11C also triggered strong induction of apoptosis as determined by the increase in fluorescence of the Incucyte caspase 3/7 dye that turns fluorescent by caspase cleaving during induction of apoptosis (**Figure 7F**). On the other hand, in this *in vitro* assay the untransformed MCF10A cell line was affected by the Warbicin**^®^** compounds in a similar way as the MCFA10 cell line transformed with *H-RAS^V12^* (**Supplementary Figure 10**). However, the growth rate of the latter was much higher than that of the untransformed cells, suggesting a possibly greater inhibitory potential on the transformed cells under appropriate conditions. These results suggest that the Warbicin^®^ compounds have a broad inhibitory effect on the proliferation of cancer cells.

### Characterization of WBC-15C, -4C and -11C for the kinetics of glucose consumption inhibition in cancer cells

**Figure 8:**
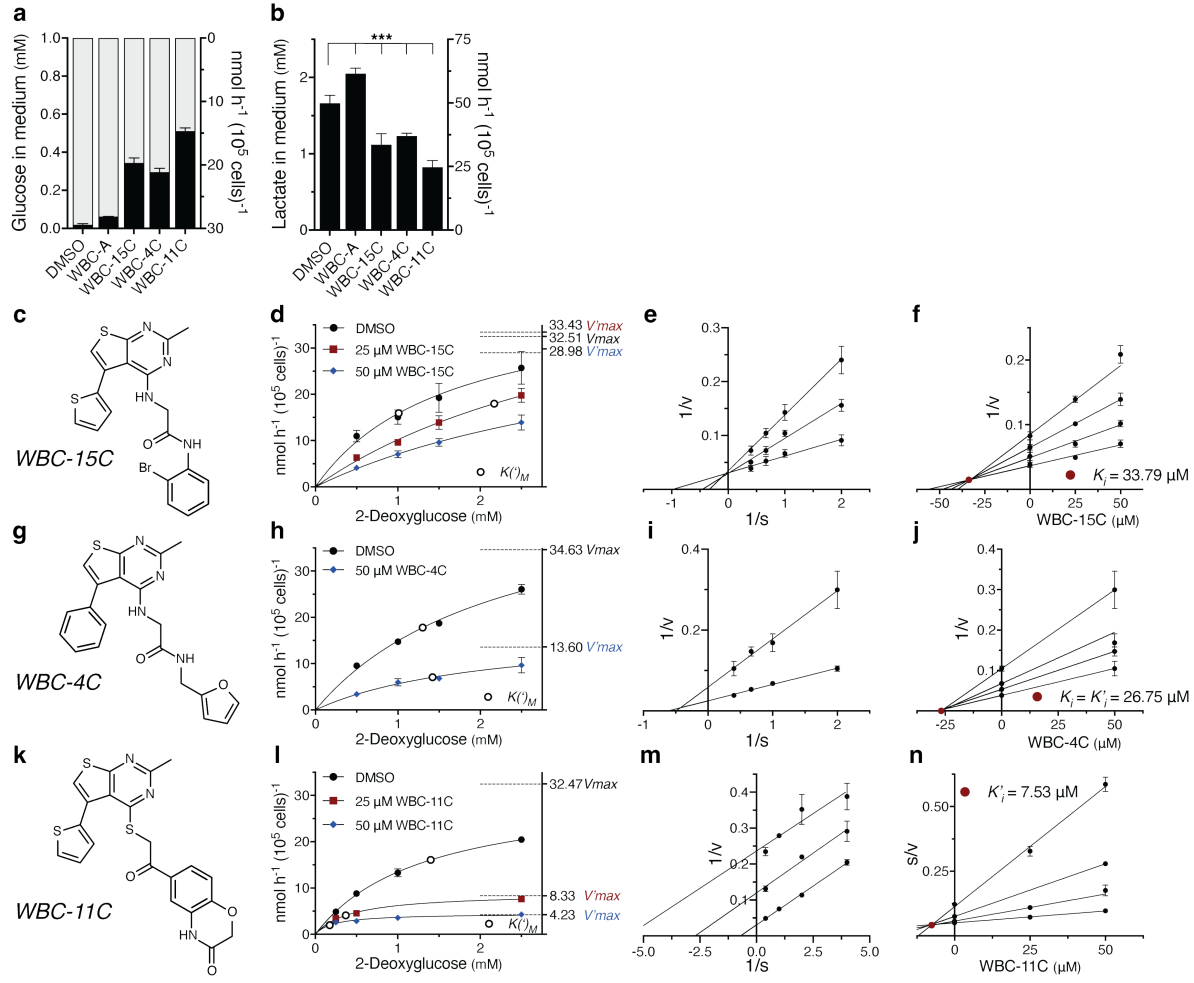
Kinetic characterization of 2-deoxyglucose transport inhibition in A549 lung adenocarcinoma cells by WBC-15C, -4C and -11C. Inhibition of **(A)** glucose consumption and **(B)** lactate accumulation in the medium by 25 µM of WBC-A, -15C, -4C and -11C in comparison with the DMSO control for A549 cells incubated in medium supplemented with 1 mM glucose for 8 (h) **(C,D)** Graphical representation of competitive and uncompetitive inhibition, respectively. Molecular structures of WBC-15 **(C)** WBC-4C **(G)** and WBC-11C **(K)**. Kinetic analysis of 2-deoxyglucose uptake inhibition in A549 cells in the absence or presence of 25 µM or 50 µM WBC-15 **(D)**, -4C **(H)** and -11C **(L)**. Corresponding *V(‘)max* (dashed lines) and *K(‘)_M_* (open circles) values are indicated. Lineweaver-Burk plot analysis, shown in **(E, I, M)**, using the data of **(D, H, L)**, respectively, for determination of mode-of-inhibition. Inhibitor constants (closed red circles) were estimated using Dixon plot analysis for WBC-15C **(F)** and WBC-4C **(J)**, whereas the Cornish-Bowden plot was applied for WBC-11C **(N)**. Significance was determined by one-way ANOVA followed by Dunnett’s multiple comparisons test (***, *p* <.001).

Our next aim was to test the effect of the most potent Warbicin^®^ compounds identified on the aerobic fermentation of cancer cells. The latter was estimated using two parameters: the extent of glucose depletion from the medium and the accumulation of lactic acid in the medium. Although they are related to each other, they do not necessarily show exactly the same response to inhibitory drugs. This was also observed in our experiments, but the deviations were minor. The compounds WBC-15C, -4C and -11C at 25 µM inhibited glucose consumption and lactate production in A549 lung adenocarcinoma cells with increasing efficiency in this order, while WBC-A did not cause inhibition within the chosen time period in these assays (**Figures 8A, B**). The same was observed for glucose consumption and lactate production in KMS-12-PE multiple myeloma cells, with largely similar efficiency as in the A549 cells (**Supplementary Figure 11**). We next characterized the kinetics of 2-deoxyglucose uptake inhibition by the Warbicin**^®^** compounds in A549 lung adenocarcinoma cells. The molecular structures of WBC-15C, -4C and -11C are shown in **Figures 8C, G, K**, respectively. The inhibition of 2-deoxyglucose uptake in A549 lung adenocarcinoma cells by 50 µM WBC-15C, -4C or -11C compared to the DMSO control is shown in **Figures 8D, H, L**, respectively. Lineweaver-Burk plot analysis revealed a different type of inhibition for the three compounds, competitive for WBC-15C (**Figure 8E**), noncompetitive for WBC-4C (**Figure 8I**) and uncompetitive for WBC-11C (**Figure 8M**). Using Dixon plot analysis, we determined a *K_i_* of 33.79 μM for WBC-15C (**Figure 8F**) and a *K_i_* (= *K’_i_*) of 26.75 μM for WBC-4C (**Figure 8J**). Since the Dixon plot cannot be used for uncompetitive inhibition, we used Cornish-Bowden plot analysis to determine the *K’_i_* of 7.53 μM for WBC-11C (**Figure 8N**).

These results show that the Warbicin^®^ compounds effectively inhibit aerobic fermentation in cancer cells and the different kinetics of inhibition with the analogs suggest a complex target.

### Evaluation of Warbicin^®^ toxicity in mice

We have evaluated the toxicity of WBC-A, WBC-15C, WBC-4C and WBC-11C in nude mice by daily intraperitoneal injection with the doses of 5, 10 and 20 mg/kg for each condition. Three mice were used for each dose. There was no significant loss in body weight over a period of 20 days, except for a small transient drop with WBC-11C at the doses of 10 and 5 mg/kg around day 5 and 11, respectively, and for one single mouse treated with 20 mg/kg WBC-4C that was sacrificed at day 19 because of 20% weight loss (**Supplementary Figure 12**). No obvious behavioral changes were detected and as such the mice were sacrificed at day 20. Autopsy of the sacrificed mice revealed one conspicuous deviation. Based on visual inspection, more adipose tissue was consistently present compared to the control mice receiving only the vehicle. This was observed with all compounds at all tested concentrations, except for a few mice belonging to the 20 (one mouse), 10 (one mouse) and 5 mg/kg (two mice) conditions of WBC-4C, respectively, and one mouse treated with 5 mg/kg WBC-11C. At the highest concentrations of WBC-15C and WBC-11C, adipose tissue was also observed in between the intestines. No other abnormalities were observed in mice treated with WBC-A or WBC-4C.

At 20 mg/kg WBC-15C and at 20 and 10 mg/kg WBC-11C, toxic effects were seen in the liver and other organs (i.e. enlarged liver and intestines, white spots on the liver and fluid in the abdomen) which was not observed at the lower concentrations of these compounds tested. No significant difference could be detected in the blood glucose level compared to the control in sera samples collected post-mortem (**Supplementary Figure 13**). Moreover, determination of the AST/ALT ratio from the same samples, to assess liver toxicity, did not reveal any significant deviation from the control (**Supplementary Figure 14**). Histological analysis of the kidneys and livers derived from the mice treated with the inhibitors showed no relevant alteration of organ morphology. The kidney structure at the level of the glomerules and tubules was not affected by the treatment, similarly there was no sign of inflammatory processes suggesting the absence of damage to the kidneys. The livers were also well preserved in terms of morphology of hepatic lobules and hepatic parenchyma. Of note, contrary to the fat tissue accumulation revealed during autopsy, there was no macro- or microvesicular steatosis present in the liver tissues. There were no signs of acute and/or chronic hepatitis or fibrosis. Some hepatocytes presented mild hydropic degeneration, a focal necrosis with some scattered inflammatory cells, but overall the histopathological analysis indicates absence of structural damage in the liver and suggests that the treatment with the Warbicin**^®^** compounds has no adverse effect on liver function.

These results show that the Warbicin^®^ compounds are well tolerated by mice in the concentrations used and that they are apparently also active *in vivo* as inhibitors of systemic glucose uptake as indicated by the pronounced increase in adipose tissue caused by all the compounds.

## Discussion

A major challenge in the design of drugs that target the Warburg effect is that the transporters and enzymes of glycolysis and likely also their basic regulation mechanisms are essentially the same in cancer and non-cancer cells. On the other hand, the Warburg effect is a nearly universal feature of cancer cells and the identification of drugs that preferentially target the overactive glycolysis in cancer cells might therefore have very broad applicability in cancer chemotherapy (82–84). Drugs that target metabolic enzymes for instance in aerobic glycolysis can likely enhance the efficacy of therapeutic agents like chemotherapy and radiotherapy (83, 85–87).

In this work, we have identified a novel class of drugs that target the Warburg effect. The lead compound, WBC-A, was isolated in a screen with the yeast *tps1Δ* strain, which has such an overactive influx of glucose and other fermentable sugars into glycolysis that it causes sugar phosphate hyperaccumulation and lethality. WBC-A restored the growth of the *tps1Δ* mutant on low glucose concentrations, consistent with inhibition of the overactive glucose uptake but still allowing basal glucose uptake to occur. This fits in principle with the goal of counteracting the Warburg effect of overactive glucose uptake and metabolism in cancer cells without compromising basal glucose metabolism in non-cancerous, healthy cells. We next showed that WBC-A and several analogs inhibit glucose uptake both in yeast cells and in mammalian cells, making it the first drug family that inhibits the glucose transporters of the two cell types.

Consistent with the critical importance of the Warburg effect for the proliferation of cancer cells, WBC-A and its analogs inhibited the proliferation of several types of cancer cells. As has generally been observed with inhibitors of aerobic glycolysis, also untransformed cells were inhibited but their growth rate is much slower than that of transformed cells. Future research will have to determine whether appropriate conditions can be found in which the Warbicin^®^ compounds preferentially affect cancer cell growth or survival versus growth or survival of nontransformed cells. Treatment with Warbicin^®^ compounds could make cancer cells more sensitive to other anticancer treatments as has been observed for many other inhibitors of aerobic glycolysis (61, 64–66). Interestingly, the Warbicin^®^ compounds caused enhanced adipose tissue formation when administered *in vivo* in mice, without causing significant toxic effects at low to moderate concentrations. This shows that they are truly active *in vivo* in the concentrations applied. The enhanced fat accumulation is also consistent with systemic inhibition of glucose uptake.

The Warbicin^®^ compounds have an adenine-like moiety in their structure suggesting that they may act as an ATP substitute. Although WBC-A had no effect on hexokinase activity *in vitro* in cell extracts, inhibition of glucose uptake in yeast cells was dependent on the presence of an active hexokinase. We also showed that in yeast hexokinase physically interacts with the Hxt7 glucose transporter both *in vitro* and *in vivo*. This suggested that a mechanism connected with interaction between glucose uptake and phosphorylation, like transport-associated phosphorylation of glucose, might be the target of the Warbicin^®^ compounds. This would fit with the kinetics which suggest a complex target. However, we cannot exclude that the Warbicin^®^ compounds might also have one or more other relevant targets.

Based on these observations, we have made a tentative hypothesis for the possible molecular action mechanism of the Warbicin^®^ compounds. We suggest that hexokinase can reversibly interact with the glucose transporters and hydrolyze the transporter-bound ATP molecule to enhance the influx of glucose into glycolysis. The process suggested may be part of a transport-associated (vectorial) phosphorylation process of glucose by the interacting hexokinase, but the increase in glucose influx in glycolysis would not just be the result of the higher efficiency of direct metabolite channeling of incoming glucose from the carrier to the hexokinase (88), but mainly due to the relief of glucose uptake inhibition by the carrier-bound ATP. Moreover, the control of glucose uptake by hexokinase-mediated hydrolysis of transporter-bound ATP might not depend on the phosphorylation of incoming substrate, since the rate of both influx and efflux of the non-phosphorylatable 3-O-methylglucose analog was strongly increased upon cellular transformation by Rous Sarcoma Virus (89).

**Figure 9:**
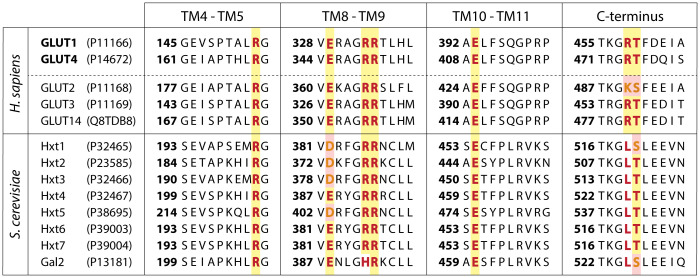
Protein sequence alignment of the ATP binding domains present in human GLUT compared with the corresponding sequences in yeast Hxt glucose transporters. The ATP binding domains described for the human GLUT1 and GLUT4 carriers are shown first, followed by alignment with the corresponding domains in human GLUT2, 3 and 14 from the same GLUT subfamily, and in the yeast Hxt transporters Hxt1, 2, 3, 4, 5, 6, 7 and Gal2 (UniProt accession numbers are shown in brackets). Fully conserved residues are indicated in red with shading in yellow, while conserved substitutions are indicated in orange with shading in pink.

The mammalian glucose carriers GLUT1 and GLUT4 are known to have a cytosolic ATP-binding domain, in which the bound ATP molecule inhibits to a certain extent glucose uptake by the carrier, leaving a residual level of glucose transport active (30, 32, 90–92). ADP also binds into the ATP-binding pocket but is unable to inhibit glucose uptake (29, 93). Non-hydrolyzable analogs of ATP cause similar inhibition of glucose uptake by GLUT1, indicating that inhibition is a direct consequence of ATP binding to GLUT1 and does not require ATP utilization as a source of energy (94). ATP binds to a Walker B motif located at the cytoplasmic loop between TM8 and TM9 in GLUT1 (33) and the same ATP binding domain is present in GLUT4 (35). ATP binding causes a constriction in the glucose transport channel, thereby lowering glucose uptake (94). The R349, R350, R474, T475 and E409 residues in GLUT4, which are fully conserved in GLUT1, are responsible for ATP binding, which is controlled by the proton-sensitive, intracellular salt bridges E329-R333/R334 in GLUT1 and E345-R349/R350 in GLUT4. The latter salt bridge network is proposed to switch upon ATP binding to the E345-R169-E409 salt bridge network (31, 33, 34). We show that all these residues are largely conserved in the yeast Hxt transporters (**Figure 9**), making it very likely that they share the same ATP-binding domain and similar regulation of glucose uptake by the bound ATP molecule as the human GLUT carriers. Up to now, however, ATP binding to yeast Hxt carriers has not been experimentally verified.

Previous work both in yeast and mammalian cells has provided indirect evidence for interaction between the glucose carriers and the glucose phosphorylating enzymes. Evidence for the occurrence of transport-associated phosphorylation was provided both in yeast (47–52) and in mammalian cells (55, 56). It has remained controversial, possibly because it is likely reversible rather than permanent as in the bacterial PTS system (46), and thus more difficult to demonstrate unequivocally. In the present work, we have provided evidence, both *in vitro* and *in vivo*, for a physical interaction between yeast Hxt7 and Hxk2, further supporting the possible occurrence of transport-associated phosphorylation in yeast. Conditional transport-associated phosphorylation of glucose might serve to adjust glucose influx into glycolysis according to the ATP level in the cells, being reduced when ATP is high and enhanced when ATP is low. It might also be an extension of the feedback inhibition of Tre6P and Glu6P on yeast and mammalian hexokinase, respectively. By inhibiting transporter-hexokinase interaction when Tre6P or Glu6P are high, glucose uptake would be reduced and thus *in vivo* hexokinase activity further reduced.

We have now shown that surprisingly WBC-A inhibits glucose and fructose uptake, and to a lower extent galactose uptake, in yeast in a way that is dependent on the presence of an active glucose phosphorylating enzyme. This is highly unexpected and has apparently never been reported previously for any sugar transport inhibitor. Future work will have to show whether the dependency on glucose phosphorylation also applies to WBC-A inhibition of the mammalian glucose carriers. We have now confirmed that classical inhibitors of the mammalian GLUT carriers have no effect on the yeast Hxt carriers. Hence, the Warbicin^®^ compounds are the first drugs that inhibit glucose uptake both in yeast and mammalian cells and a common target mechanism appears likely. This is further supported by the observation that the mammalian GLUT1 and GLUT4 carriers were also inhibited by WBC-A when expressed in yeast (**Figures 6A, B**). This suggests a broad action of this type of compound on the glucose transporters of the MFS family, that is different from the action mechanisms of the classical GLUT inhibitors.

WBC-A and nearly all its active structural analogs share a common adenine-like moiety, which appears to be essential for activity in inhibiting glucose uptake. Hence, the Warbicin^®^ compounds may act as partial structural analogs of ATP and replace the ATP molecule bound to the intracellular domain of the glucose transporters. This would inhibit glucose uptake in a similar way as shown previously for other non-hydrolyzable ATP analogs like adenosine (92) and caffeine (95). It would also preserve at least some residual glucose transport activity as in ATP-bound glucose carriers. Since hexokinase would not be able to relieve the inhibition by the Warbicin^®^ compound, glucose uptake in yeast *tps1Δ* cells and in cancer cells would be permanently reduced, similar to the inhibition of glucose uptake caused by carrier-bound ATP. This would counteract the aberrant increase in transporter activity, which is suggested to be caused by permanent interaction with hexokinase in yeast *tps1Δ* and cancer cells. This hypothesis is able to explain our observation that the inhibitory effect of the Warbicin^®^ compounds is largely dependent on the presence of a glucose phosphorylating enzyme. In the absence of hexokinase, all glucose carriers would have permanently ATP bound in the cytosolic domain and its replacement by a Warbicin**^®^** compound therefore would have little further inhibitory effect. In the presence of active hexokinase, on the other hand, at least part of the carriers would have ADP bound instead of ATP, and replacement of the ADP molecule by the non-hydrolyzable Warbicin**^®^** compound would increase inhibition of glucose uptake by the carrier. The high hydrophobicity of the Warbicin^®^ compounds fits with binding into the ATP-binding site of the intracellular domain of the membrane-localized glucose transporters. Their high hydrophobicity may confine their binding to these highly hydrophobic membrane proteins and minimize interaction with the many ATP-binding soluble proteins in cells. This could explain why we did not observe any signs of major toxicity of the Warbicin**^®^** compounds in appropriate concentrations in healthy mice.

It is tempting to speculate that the overactive influx of glucose into glycolysis in cancer cells and in yeast *tps1Δ* cells may be due in both cell types to defective control of the reversible interaction between the glucose transporters and the glucose phosphorylating enzymes, leading to permanent hexokinase-transporter interaction. This would cause persistent breakdown of the inhibitory ATP molecule into non-inhibitory ADP bound to the glucose transporters and thus cause persistent overactive glucose uptake and influx into glycolysis. Since *tps1Δ* cells lack feedback inhibition by Tre6P on hexokinase (40), this is bound to cause the dramatic deregulation of glycolysis observed, consisting of continuous overactive glucose influx into glycolysis, hyperaccumulation of sugar phosphates, depletion of ATP and free phosphate, and lowering of the intracellular pH causing blockage of glycolysis at the level of GAPDH and ultimately apoptosis and cell death. Since cancer cells, on the other hand, still have at least to some extent feedback inhibition by Glu6P on hexokinase (11), it would also lead to permanently overactive but still controlled glucose influx into glycolysis, creating the Warburg effect, leading to stimulation of uncontrolled cellular proliferation. Enhanced interaction of hexokinase with the glucose transporters is consistent with the observation that upon transformation with Rous Sarcoma Virus the intracellular glucose level drops in spite of a higher glucose uptake rate and glycolytic flux (96). The cause of the proposed aberrant interaction between glucose transporters and glucose phosphorylating enzymes remains unclear. It could be triggered by oncogene signaling and supported by aberrant overexpression of glucose carriers and hexokinases. The importance of glucose carrier – hexokinase interaction for the glucose growth defect of the *tps1Δ* strain is supported by the observation that deletion of *HXK2* completely abolishes both the glucose growth defect and the deregulation of glycolysis (44) and that restraining hexokinase in the nucleus lowers the glucose sensitivity of *tps1Δ* cells (**Figure 5C**).

With the single exception of WBC-55A, all compounds that rescued the *tps1Δ* strain on low glucose had a different modification of the sulphur-linked side group attached to the adenine-like structure (**Supplementary Table 1**), while other variations completely abolished the rescue of the *tps1Δ* mutant on low glucose (**Supplementary Table 2**). WBC-55C was highly active in rescuing growth of *tps1Δ* cells on glucose. However, it did not prevent hyperaccumulation of sugar phosphates, at least not in the short term, suggesting that its main target might be located downstream in the signaling pathway between the glycolytic deregulation and the induction of apoptosis.

In this work we aimed to identify small molecules that would inhibit the overactive glucose uptake in cancer cells without compromising basal glucose uptake in healthy cells, and thus specifically targeting the Warburg effect. The new Warbicin^®^ family of drug compounds might in the appropriate concentration fulfill this requirement. A wealth of evidence is available that cancer cells are highly dependent on the hyperactive flux through glycolysis of the Warburg effect, and that inhibition of glycolysis enhances the sensitivity of cancer cells to different types of cancer treatments, such as chemotherapeutics and radiation (84, 85, 97). In that respect, also the GLUT transporters have been proposed as attractive targets for anticancer drug treatment (98), especially since glucose uptake may have a major role in rate-control of glycolytic flux in cancer cells (9, 11). Several reports have documented that inhibitors or other mechanisms of GLUT downregulation render cancer cells more drug-sensitive (68, 99–106). Up to now, however, no drugs have been available that preferentially act on the hyperactive glucose flux in cancer cells without compromising glycolytic flux in healthy cells.

In conclusion, we have identified a new family of drugs that reduce glucose influx into glycolysis and constitute attractive candidates for specifically targeting the Warburg effect. The effectiveness of the Warbicin^®^ compounds both on the yeast and mammalian glucose transporters and their unusual dependency on active glucose phosphorylation, seems to indicate that they target a novel unknown mechanism controlling glucose influx into glycolysis. We suggest that reversible hexokinase interaction with the glucose transporters controls glucose uptake through hydrolysis of the carrier-bound ATP molecule and that the Warbicin^®^ compounds exert their inhibitory effect by replacing the ATP molecule and thus preventing aberrant hexokinase mediated stimulation of glucose uptake, presumed to occur in yeast *tps1Δ* cells and in cancer cells.

## Data Availability

The original contributions presented in the study are included in the article/**Supplementary Material**. Further inquiries can be directed to the corresponding author.
