# Supplementary material for: The novel family of Warbicin® compounds inhibits glucose uptake both in yeast and human cells and restrains cancer cell proliferation
Source: Front Oncol. 2024 Aug 22;14:1411983. doi: 10.3389/fonc.2024.1411983 (PMC11374660; doi:10.3389/fonc.2024.1411983)
Supplement: Supplementary file 1 [file DataSheet1.docx]

**Supplementary Materials**

**Supplementary Figures**

**Supplementary Fig. 1 Kinetic characterization of WBC-A inhibition of glucose uptake in yeast *tps1∆* cells. a.** Kinetic analysis of glucose uptake in yeast *tps1∆* cells in the absence or presence of 25 µM WBC-A or 50 µM WBC-A. Corresponding *V(‘)max* (dashed lines) and *K(‘)_M_* (open circles) values are indicated. **b.** Lineweaver-Burk plot analysis for the determination of mode-of-inhibition. **c.** Corresponding Dixon plot analysis for estimation of the *K_i_* (closed red circle) and *K’_i_* (closed blue circle) inhibitor constants of WBC-A. For all experiments, cells were (pre)grown on Complete Synthetic medium containing 110 mM galactose.

**Supplementary Fig. 2 Hexokinase activity in extracts of wild type yeast cells is unaffected by WBC-A.** Hexokinase activity was measured in extracts of wild type yeast cells grown on 110 mM galactose. The activity in the presence of DMSO as control or 50 µM WBC-A was determined with **a.** different glucose concentrations and a fixed ATP concentration of 5 mM and **b.** different ATP concentrations and a fixed glucose concentration of 5 mM.


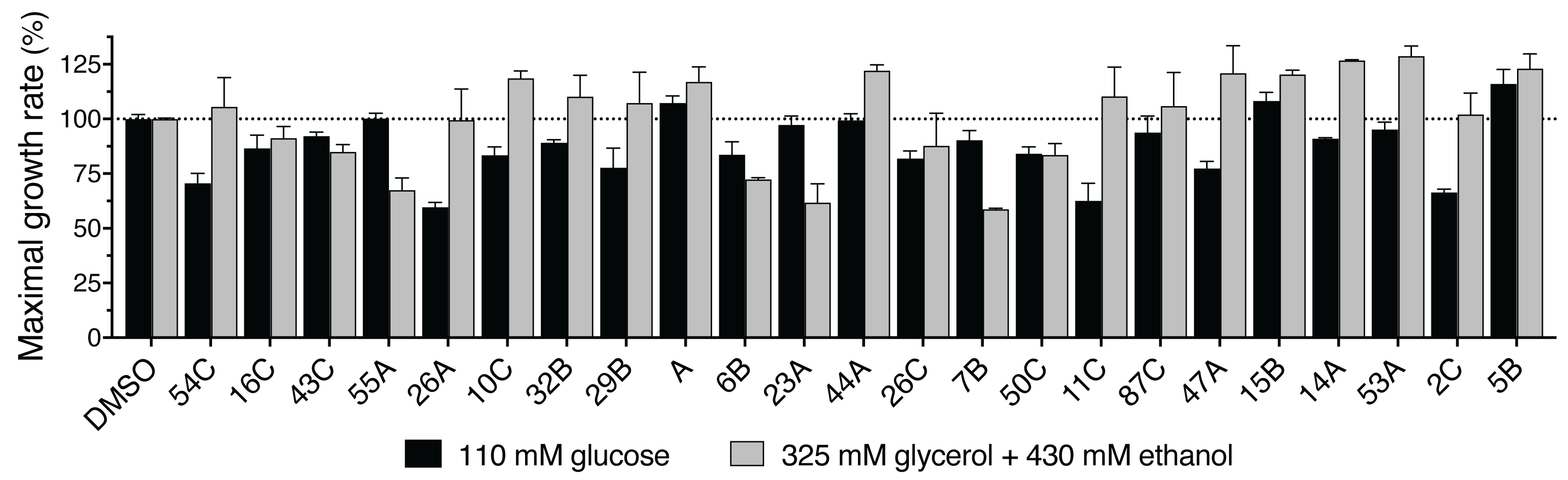


**Supplementary Fig. 3 General effect of WBC compounds on wild type yeast growth.** WBC compounds that rescued growth of yeast *tps1∆* cells on glucose were compared for their effect at 100 µM on wild type yeast growth on 110 mM glucose and 325 mM glycerol + 430 mM ethanol. Maximal growth rates were determined after 2 days of growth in Complete Synthetic liquid medium.

**Supplementary Fig. 4 WBC-55A has a different structure and action mechanism.** **a.** Molecular structure of WBC-55A. Metabolic profiles for **b.** Glu6P and **c.** Fru1,6bisP accumulation after addition of glucose. At time point zero, 2.5 mM glucose was added to yeast *tps1∆* cells in the absence or presence of 100 µM WBC-A or 100 µM WBC-55A. Inhibitors were added at -10 min.

**Supplementary Fig. 5 The free C-terminal part of Citrine does not spontaneously assemble with yeast Hxt7-NCitr.** Fluorescence microscopy images to assess spontaneous BiFC self-assembly. Hxt7-Citrine yeast cells transformed with the empty plasmid show the expected localization of Hxt7 at the plasma membrane (left image). Hxt7-NCitr yeast cells transformed with vector-expressed full-length Citrine show correct expression of Citrine in the cytosol (middle image). Hxt7-NCitr yeast cells transformed with vector-expressed CCitr do not show any fluorescence (right image: fluorescence and DIC). Cells were grown on YP medium supplemented with 325 mM glycerol and 430 mM ethanol.

**
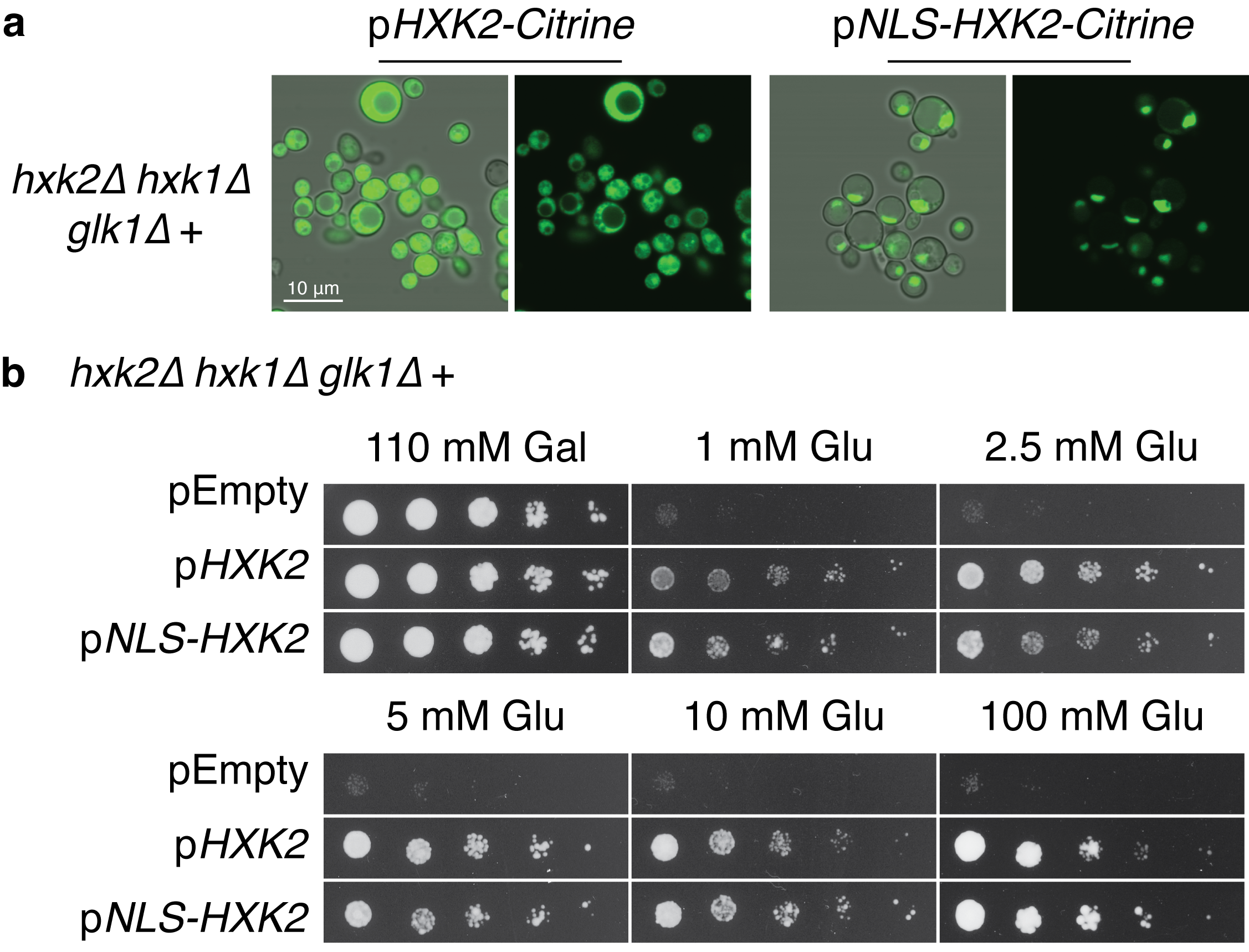
**

**Supplementary Fig. 6 Cytosolic and nuclear localized hexokinase restores glucose growth of the yeast *hxk^0^* mutant. a.** Fluorescent microscopic images of *hxk2∆ hxk1∆ glk1∆* yeast cells transformed with a vector expressing either a *HXK2* or a *NLS-HXK2* allele. **b.** Spot assay for growth on 110 mM galactose or different levels of glucose of *hxk2∆ hxk1∆ glk1∆* yeast cells transformed with a vector containing either no insert, a *HXK2* or a *NLS-HXK2* allele. Pictures were taken after 3 days. For every experiment, cells were pregrown on 325 mM glycerol and 430 mM ethanol in uracil-deficient medium for plasmid retention.

**Supplementary Fig. 7 Inhibition of fructose and galactose uptake by WBC-A in yeast strains with and without corresponding functional sugar kinase activity. a.** Inhibition of 1 mM fructose uptake by *HXK2 hxk1∆ glk1∆* and *hxk2∆ hxk1∆ GLK1* cells treated with either DMSO or 50 µM WBC-A. **c.** Inhibition of 1 mM galactose uptake by wild type, *gal80∆*, *gal80∆ gal3∆*, *gal80∆ gal1∆*, *gal80∆ gal1∆ gal3∆* cells treated with either DMSO or 50 µM WBC-A. Uptake rates of **a**. and **c.** were set relative to the DMSO control (100%) in **b.** and **d.** for each strain, respectively. Significance was determined by two-way ANOVA with Sidak’s multiple comparisons test (**, *p* < .01; ***, *p* < .001; ns, non-significant).

**Supplementary Fig. 8 Effect of known mammalian glucose uptake inhibitors on glucose transport by Hxt7 and GLUT1 expressed in yeast.** Inhibition of 2.5 mM glucose uptake in *hxt^0^ gal2∆* yeast cells expressing either a *HXT7* or *GLUT1^V69M^* allele. Cells were treated with either DMSO or 25 µM of WBC-A, Fasentin, STF-31, Cytochalasin B, WZB-117 and BAY-876. Significance is determined by two-way ANOVA followed by Sidak’s multiple comparisons test (***, *p* < .001). Cells were grown on rich medium containing 60 mM maltose.

**
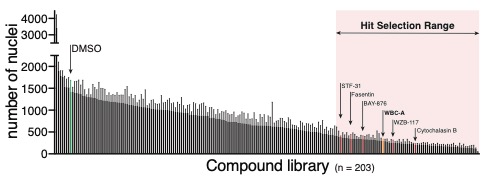
**

**Supplementary Fig. 9 Prescreening of the structural analog library of WBC-A on A549 cancer cells**. The analog library (n = 203) was screened for inhibition of growth of A549 cancer cells on RPMI medium supplemented with 1 mM glucose. DMSO (green bar) and WBC-A (orange bar) served as negative and positive control, respectively. In addition, the reference glucose transport inhibitors STF-31, Fasentin, BAY-876, WZB-117 and Cytochalasin B were included and indicated in red. Compounds were added at 50 µM concentration. Number of cells was determined by counting nuclei stained by Hoechst after three days of growth.

**
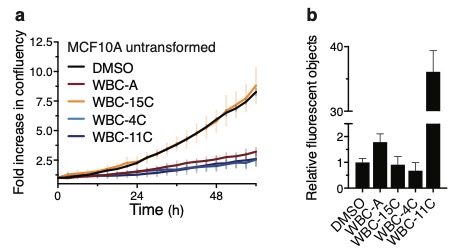
**

**Supplementary Fig. 10 Structural analogs of WBC-A inhibit cell proliferation and WBC-11C induces apoptosis in the untransformed MCF10A cell line. a.** Growth curve of the MCF10A breast epithelial cell line on 1 mM glucose. Cells were treated with either DMSO, or 25 µM of WBC-A, WBC-15C, WBC-4C or WBC-11C. Growth was based on increase in confluency as determined by the Incucyte software. **b.** Relative fluorescent object counts originating from apoptosis induction in MCF10A cells growing on 1 mM glucose, as determined by the Incucyte software. Cells were treated with either DMSO or 25 µM of WBC-A, -15C, -4C or -11C. Fluorescent object counts after three days were corrected for total confluency percentage and normalized to the DMSO control.

**Supplementary Fig. 11 Warbicin^®^ compounds inhibit glucose consumption and lactate production in the KMS-12-PE multiple myeloma cell line.** Glucose consumption (**a.**) and lactate production (**b.**) are shown for KMS-12-PE cells incubated in RPMI medium supplemented with 1 mM glucose for 8 h. Significance was determined by one-way ANOVA with Dunnett’s multiple comparisons test (***, *p* < .001).

**Supplementary Fig. 12 Evaluation of Warbicin^®^ toxicity in mice: weight loss.** Weight loss was determined during 20 days in nude mice treated with WBC-A, WBC-15C, WBC-4C and WBC-11C by daily intraperitoneal injection. **a.** 20 mg/kg, **b.** 10 mg/kg and **c**. 5 mg/kg. Three mice were used for each dose. Standard deviation is shown.

**Supplementary Fig. 13 Evaluation of Warbicin^®^ toxicity in mice: blood glucose level.** The blood glucose level was determined from sera samples collected post-mortem after 20 days of treatment with WBC-A, WBC-15C, WBC-4C and WBC-11C by daily intraperitoneal injection. **a.** 20 mg/kg, **b.** 10 mg/kg and **c.** 5 mg/kg. Three mice were used for each dose. Standard deviation is shown. No significant difference (*p* > .05) between vehicle and compound treated mice was observed across all tested concentrations by applying one-way ANOVA statistical analysis.

**Supplementary Fig. 14 Evaluation of Warbicin^®^ toxicity in mice: AST/ALT ratio.** To evaluate liver toxicity, the AST/ALT ratio was determined in sera samples collected after 20 days at the end of the experiment from the nude mice treated with WBC-A, WBC-15C, WBC-4C or WBC-11C by daily intraperitoneal injection. **a.** 20 mg/kg, **b.** 10 mg/kg and **c**. 5 mg/kg. Three mice were used for each dose. Standard deviation is shown. No significant difference (*p* > .05) between vehicle and compound treated mice was observed across all tested concentrations by applying one-way ANOVA statistical analysis.

**Supplementary Tables**

**Supplementary Table 1 Overview of the molecular structures of WBC-A and structural analogs that rescued growth of the yeast *tps1∆* strain on glucose.** For every compound, both the IC_50_ value for 2.5 mM glucose transport inhibition and the minimal rescue concentration for growth of the yeast *tps1∆* strain on 2.5 mM glucose are shown. The common backbone structure is illustrated of which the R-group denotes the compound-specific side chain.

**Supplementary Table 2 Overview of the molecular structure of WBC-A analogs and their bioactivity with respect to growth rescue of the yeast *tps1∆* strain and growth inhibition of the A549 cancer cell line.** WBC-A and its structural analogs are listed with their corresponding vendor, ID-code and molecular structure. A distinction is made between compounds that could or could not rescue yeast *tps1∆* growth on 2.5 mM glucose. In addition, compounds selected from the primary A549 growth inhibitory screen and compounds with a higher IC_50_ ratio, for growth on 10 mM glucose to 1 mM glucose, compared to WBC-A are indicated.
