# Supplementary material for: The novel family of Warbicin® compounds inhibits glucose uptake both in yeast and human cells and restrains cancer cell proliferation
Source: Front Oncol. 2024 Aug 22;14:1411983. doi: 10.3389/fonc.2024.1411983 (PMC11374660; doi:10.3389/fonc.2024.1411983)
Supplement: Supplementary file 2 [file Table1.docx]

**Supplementary materials**

**Supplementary Table 1 Molecular structure of the 21 analogs of WBC-A that rescue to different extent growth on glucose of *tps1∆* cells.** For every compound, both the IC50 value for 2.5 mM glucose transport inhibition and the minimal rescue concentration for growth of the *tps1∆* strain on 2.5 mM glucose are shown. The common backbone structure is illustrated of which the R-group denotes the compound-specific side-chain.

| Name | Structure |  |  |
| --- | --- | --- | --- |
| Conserved backbone | 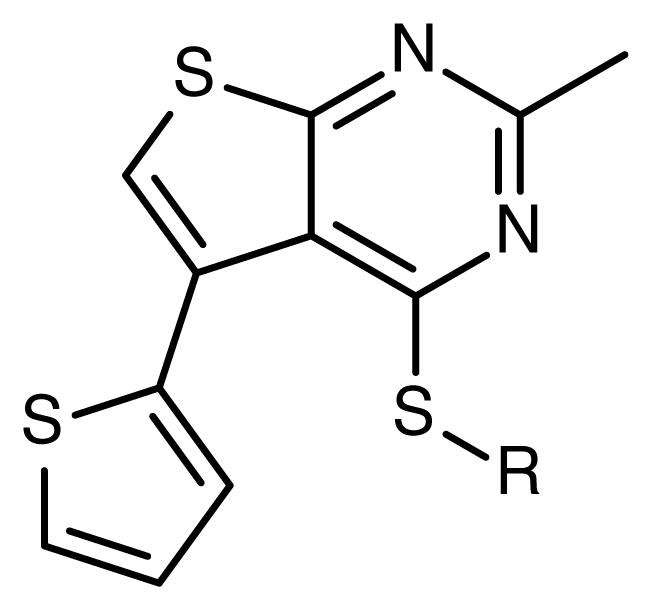 |  |  |
| R-group | Structure | IC_50_ values for 2.5 mM glucose uptake in *tps1∆* cells (µM) | Minimal rescue concentration for growth of *tps1∆* cells on 2.5 mM glucose (µM) |
| WBC-2C | 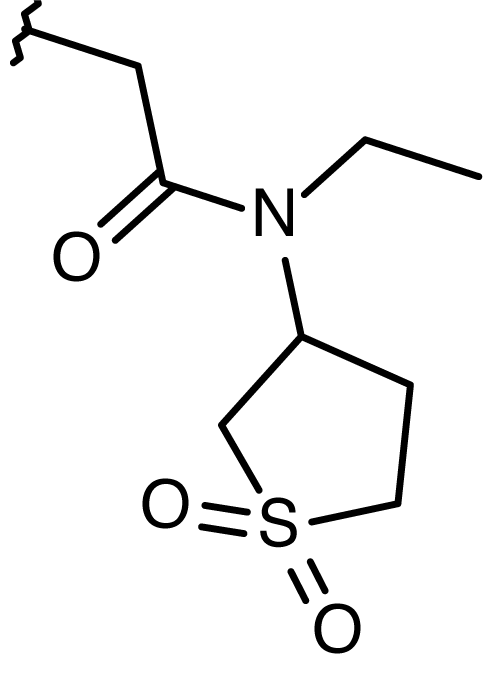 | 2.0 | 1.562 |
| WBC-26A | 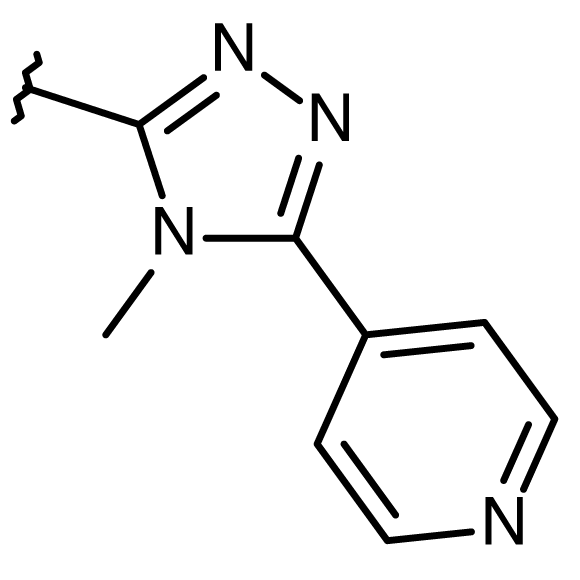 | 5.35 | 25 |
| WBC-7B | 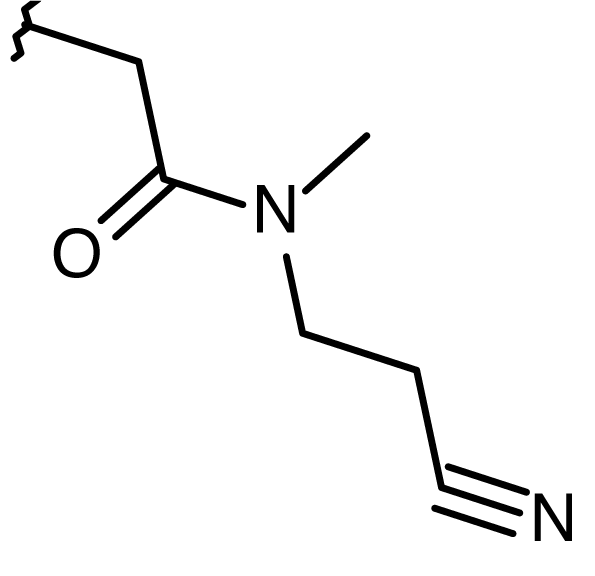 | 5.41 | 6.25 |
| WBC-23A | 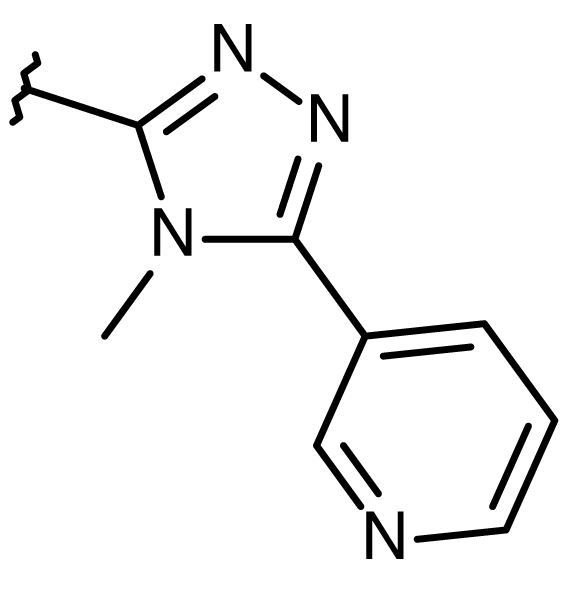 | 8.60 | 25 |
| WBC-43C | 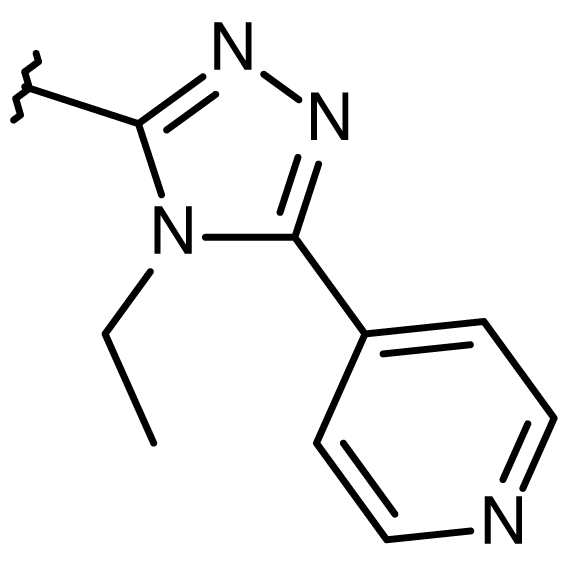 | 8.93 | 12.5 |
| WBC-29B | 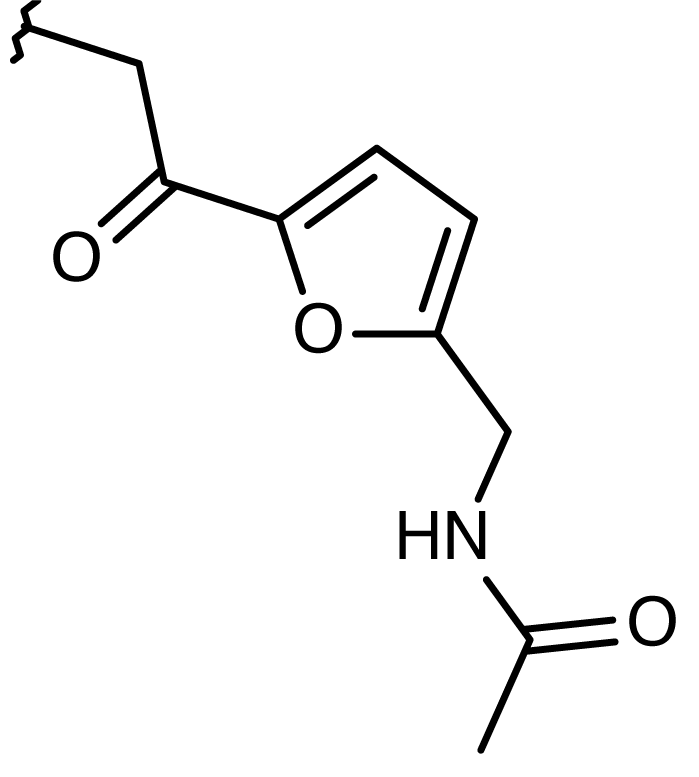 | 9.23 | 6.25 |
| WBC-6B | 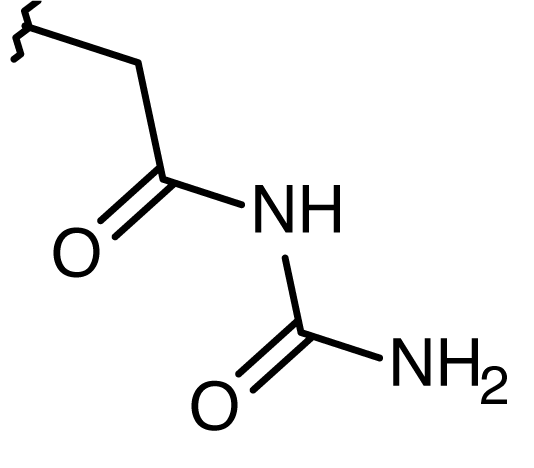 | 10.17 | 12.5 |
| WBC-87C | 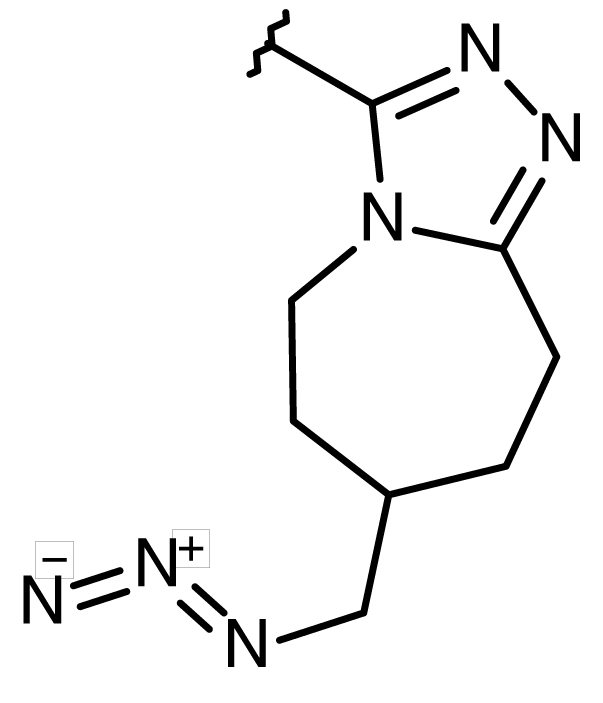 | 11.08 | 12.5 |
| WBC-47A | 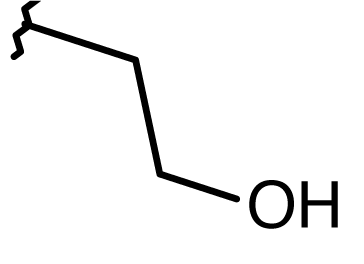 | 15.59 | 6.25 |
| WBC-A | 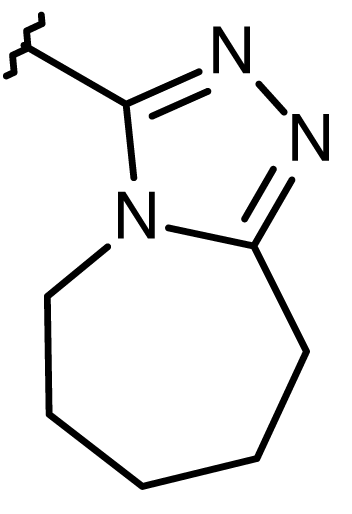 | 20.75 | 25 |
| WBC-5B | 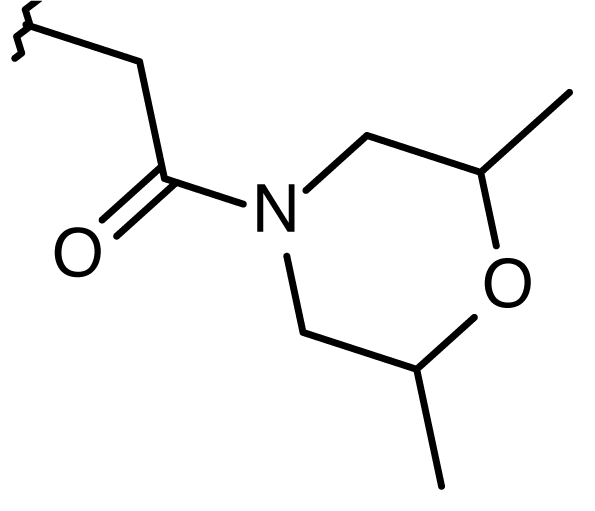 | 22.51 | 12.5 |
| WBC-26C | 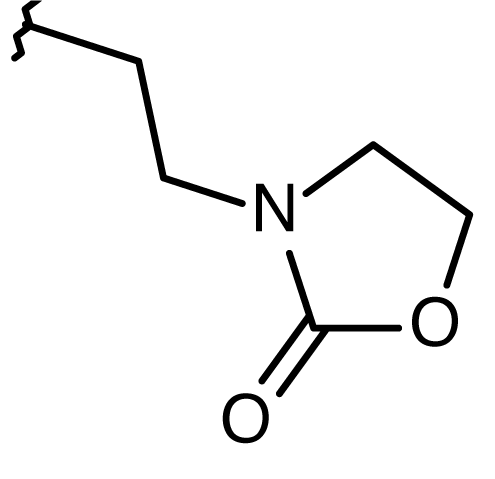 | 23.82 | 25 |
| WBC-10C | 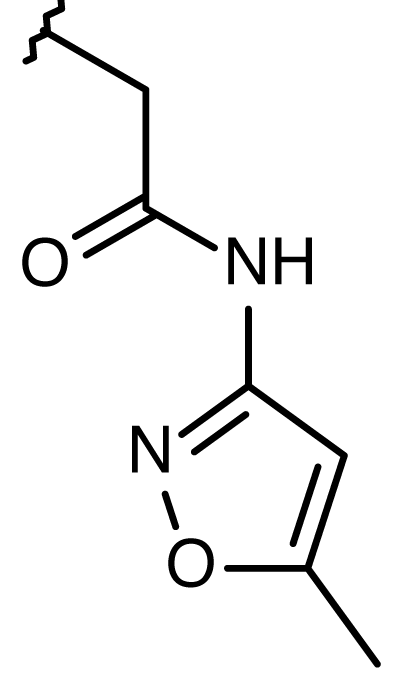 | 24.81 | 6.25 |
| WBC-53A | 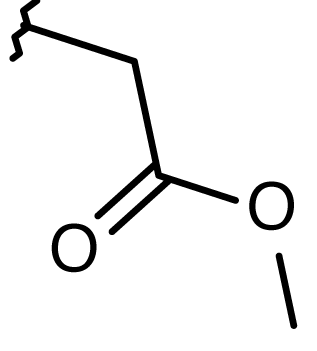 | 26.75 | 12.5 |
| WBC-14A | 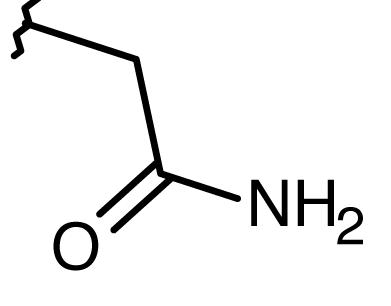 | 27.94 | 25 |
| WBC-32B | 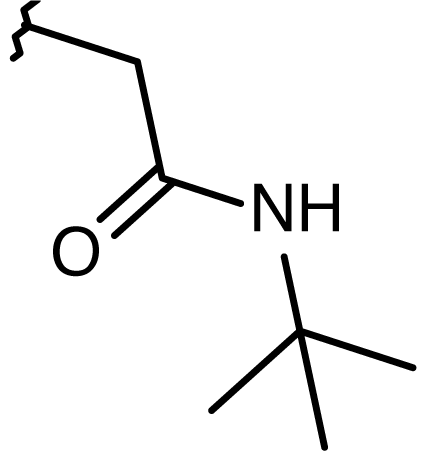 | 30.29 | 12.5 |
| WBC-15B | 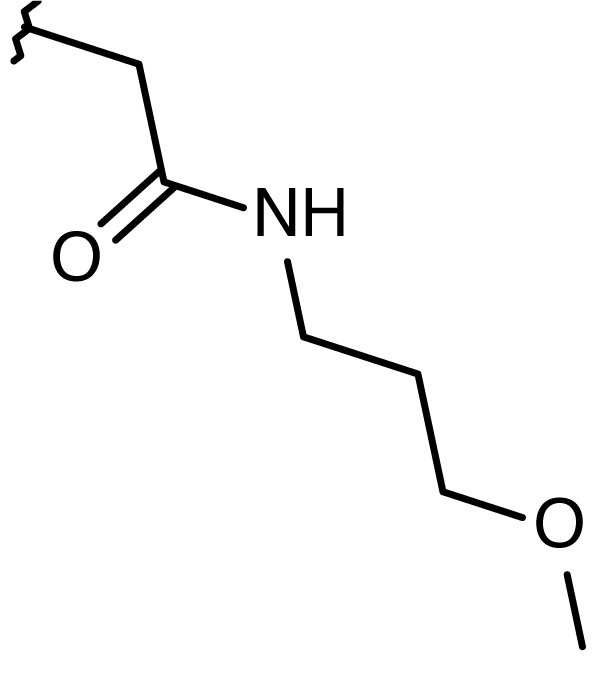 | 32.72 | 25 |
| WBC-11C | 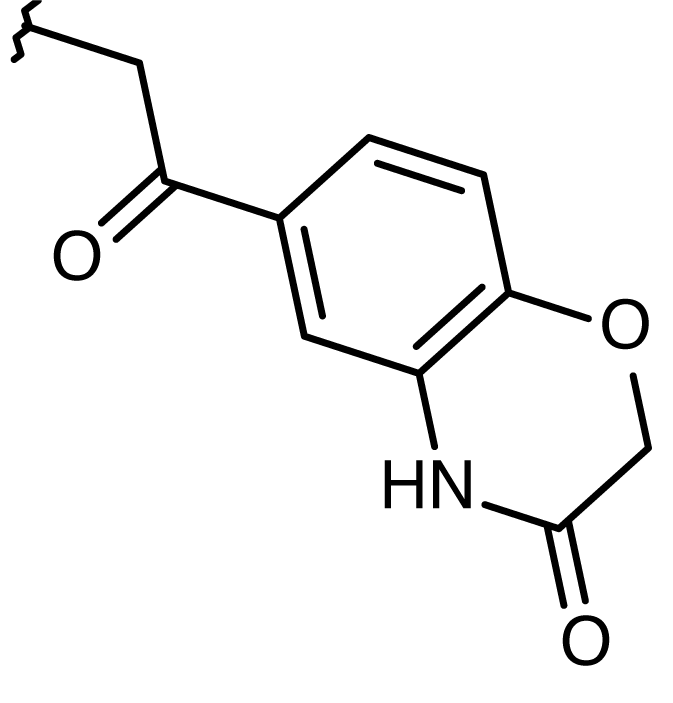 | 44.01 | 50 |
| WBC-16C | 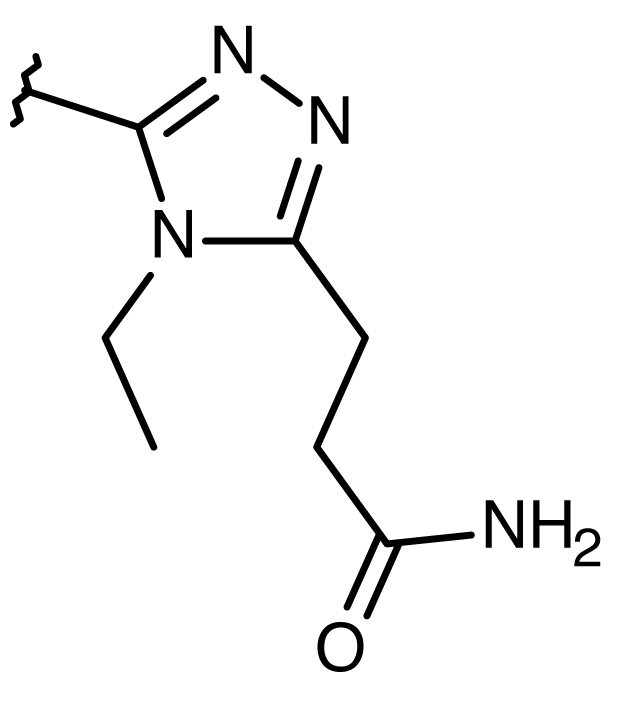 | 49.62 | 50 |
| WBC-44A | 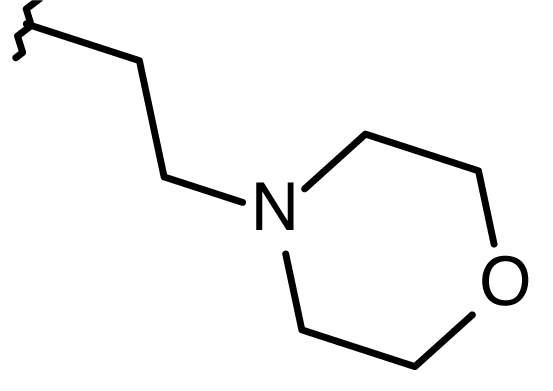 | 61.75 | 50 |
| WBC-54C | 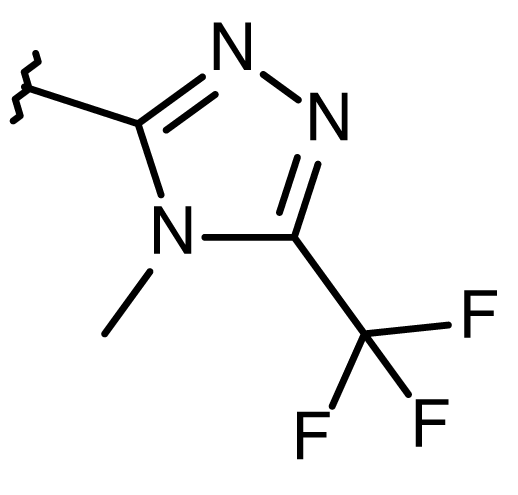 | 64.66 | 50 |
| WBC-50C | 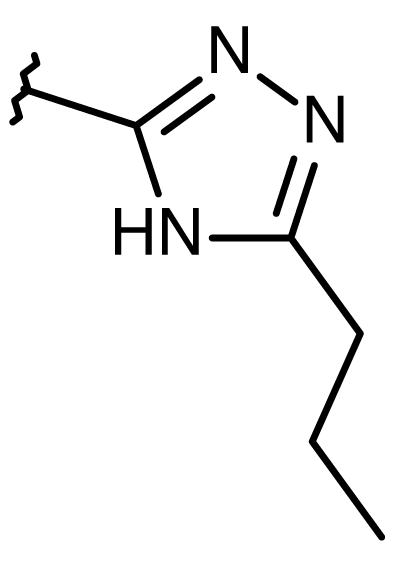 | 67.77 | 50 |
