# Supplementary material for: The novel family of Warbicin® compounds inhibits glucose uptake both in yeast and human cells and restrains cancer cell proliferation
Source: Front Oncol. 2024 Aug 22;14:1411983. doi: 10.3389/fonc.2024.1411983 (PMC11374660; doi:10.3389/fonc.2024.1411983)
Supplement: Supplementary file 3 [file Table2.docx]

**Supplementary Table 2 Overview of the molecular structure of WBC-A analogs and their bioactivity with respect to growth rescue of the *tps1∆* strain and growth inhibition of the A549 cell line.** WBC-A and its structural analogs are listed with their corresponding vendor, ID-code and molecular structure. A distinction is made between compounds that could or could not rescue *tps1∆* growth on 2.5 mM glucose. In addition, compounds selected from the primary A549 growth inhibitory screen and compounds with a higher IC50 ratio (10 mM glucose : 1 mM glucose) compared to WBC-A are indicated.

| Compound name (WBC-) | Compound vendor and code | Structure | Rescue of the *tps1*∆ strain on 2.5 mM glucose at 100 µM | Selected from primary screen for growth inhibition of A549 cells at 50 µM | Selected for having an IC_50_ ratio (10 mM glucose : 1 mM glucose) of A549 growth higher than WBC-A |
| --- | --- | --- | --- | --- | --- |
| A | Enamine,  Z85926102 | 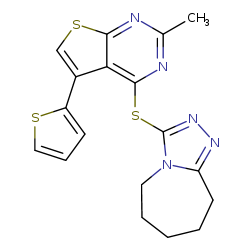 | Yes | Yes | Used as cutoff threshold |
| 1A | Enamine, Z57986816 | 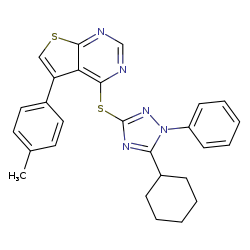 | No | No | Not tested |
| 2A | Enamine, Z85925971 | 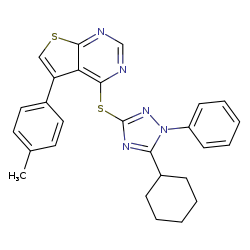 | No | No | Not tested |
| 3A | Enamine, Z85934181 | 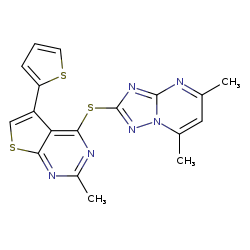 | No | No | Not tested |
| 4A | Enamine, Z2239062993 | 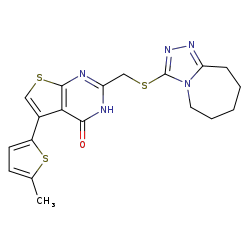 | No | No | Not tested |
| 5A | Enamine, Z31109634 | 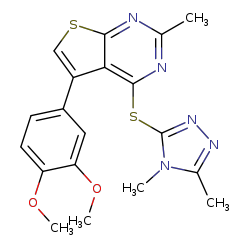 | No | No | Not tested |
| 6A | Enamine, Z21579098 | 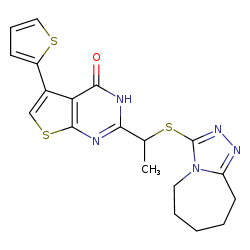 | No | No | Not tested |
| 7A | Enamine, Z212114188 | 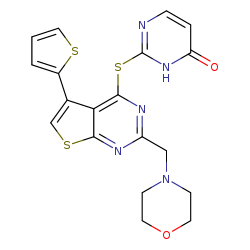 | No | No | Not tested |
| 8A | Enamine, Z198754772 | 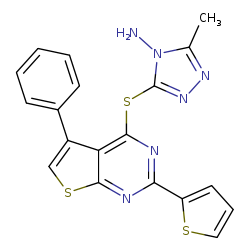 | No | No | Not tested |
| 9A | Enamine, Z2239070437 | 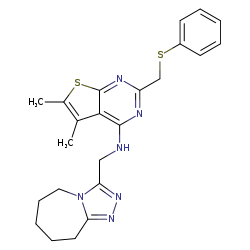 | No | No | Not tested |
| 10A | Enamine, Z234870892 | 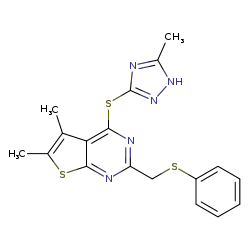 | No | No | Not tested |
| 11A | Enamine, Z193493822 | 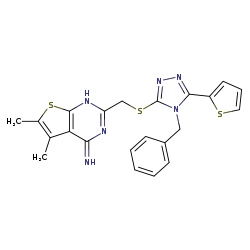 | No | No | Not tested |
| 12A | Enamine, Z199929046 | 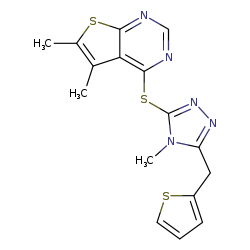 | No | No | Not tested |
| 13A | Enamine, Z2239072210 | 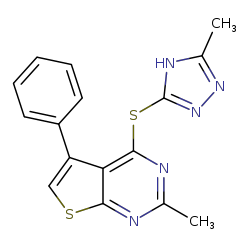 | No | No | Not tested |
| 14A | Enamine, Z16637978 | 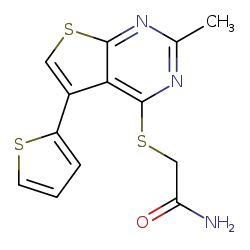 | Yes | No | Not tested |
| 15A | Enamine, Z16638026 | 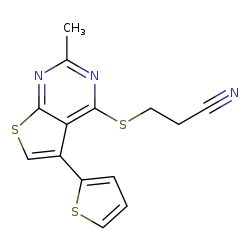 | No | No | Not tested |
| 16A | Enamine, Z31109644 | 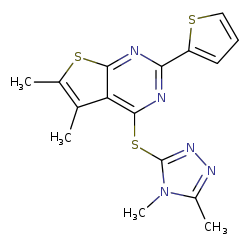 | No | No | Not tested |
| 17A | Enamine, Z275103620 | 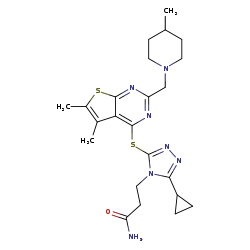 | No | Yes | Lower IC_50_ ratio |
| 18A | Enamine, Z16637998 | 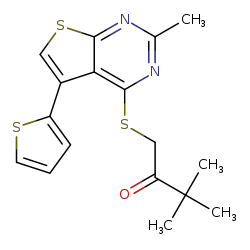 | No | Yes | Lower IC_50_ ratio |
| 19A | Enamine, Z31109564 | 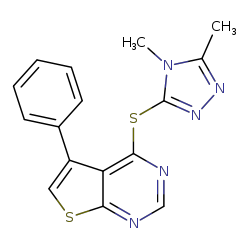 | No | No | Not tested |
| 20A | Enamine, Z199817504 | 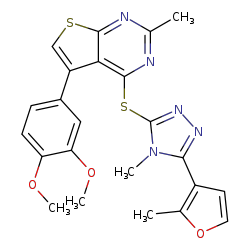 | No | Yes | Lower IC_50_ ratio |
| 21A | Enamine, Z200072422 | 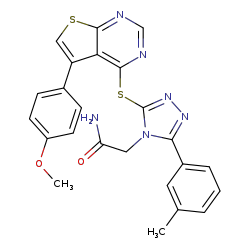 | No | Yes | Lower IC_50_ ratio |
| 22A | Enamine, Z220377504 | 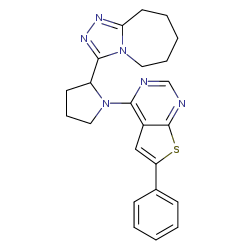 | No | No | Not tested |
| 23A | Enamine, Z199805674 | 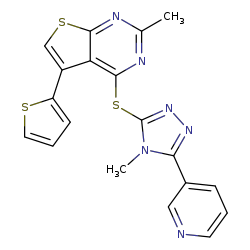 | Yes | Yes | Lower IC_50_ ratio |
| 24A | Enamine, Z198006244 | 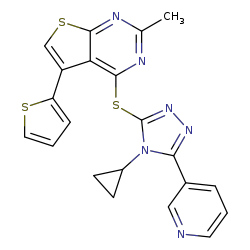 | No | Yes | Lower IC_50_ ratio |
| 25A | Enamine, Z200045206 | 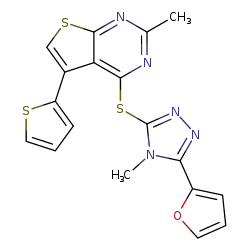 | No | No | Not tested |
| 26A | Enamine, Z199723150 | 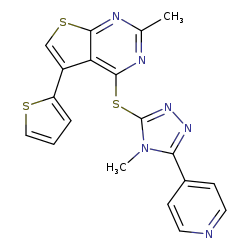 | Yes | Yes | Lower IC_50_ ratio |
| 27A | Enamine, Z200026606 | 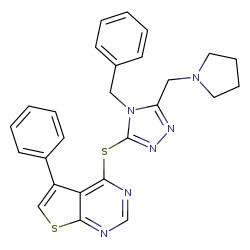 | No | Yes | Lower IC_50_ ratio |
| 28A | Enamine, Z198001832 | 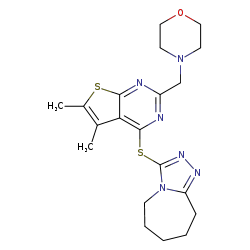 | No | No | Not tested |
| 29A | Enamine, Z64619643 | 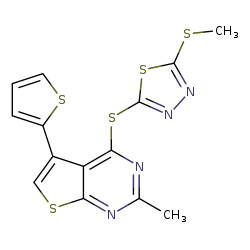 | No | No | Not tested |
| 30A | Enamine, Z31083015 | 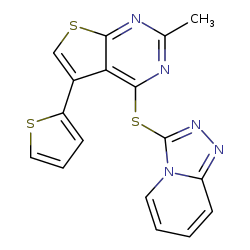 | No | No | Not tested |
| 31A | Enamine, Z198001782 | 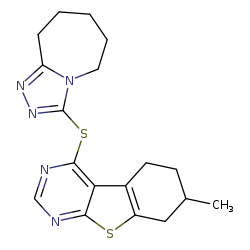 | No | No | Not tested |
| 32A | Enamine, Z200173686 | 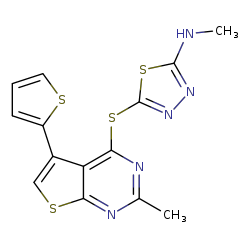 | No | No | Not tested |
| 33A | Enamine, Z645064812 | 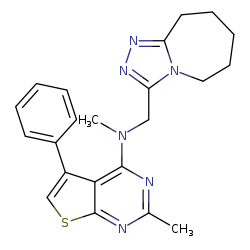 | No | No | Not tested |
| 34A | Enamine, Z645064508 | 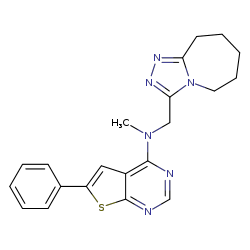 | No | No | Not tested |
| 35A | Enamine, Z193725386 | 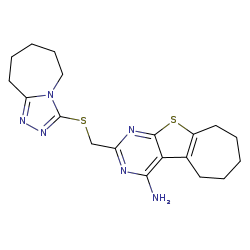 | No | Yes | Lower IC_50_ ratio |
| 36A | Enamine, Z645064922 | 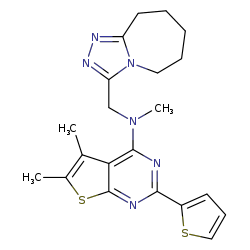 | No | No | Not tested |
| 37A | Enamine, Z1334451862 | 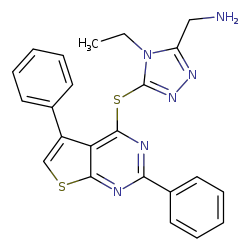 | No | No | Not tested |
| 38A | Enamine, Z361946650 | 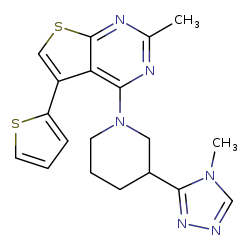 | No | No | Not tested |
| 39A | Enamine, Z31243547 | 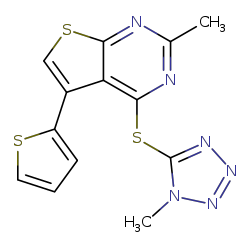 | No | Yes | Lower IC_50_ ratio |
| 40A | Enamine, Z1383881908 | 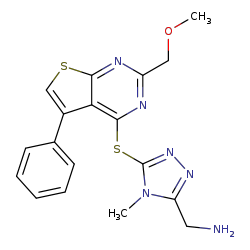 | No | No | Not tested |
| 41A | Enamine, Z1526936211 | 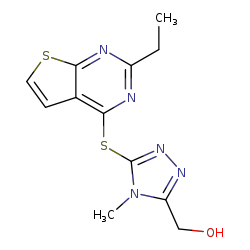 | No | Yes | Lower IC_50_ ratio |
| 42A | Enamine, Z31241654 | 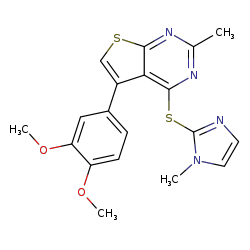 | No | Yes | Lower IC_50_ ratio |
| 43A | Enamine, Z31243444 | 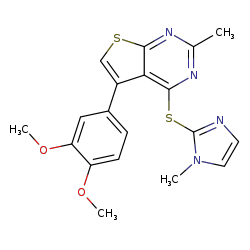 | No | Yes | Lower IC_50_ ratio |
| 44A | Enamine, Z16637947 | 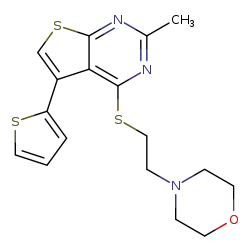 | Yes | Yes | Lower IC_50_ ratio |
| 45A | Enamine, Z16637970 | 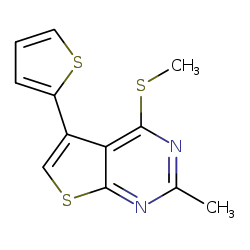 | No | No | Not tested |
| 46A | Enamine, Z31082970 | 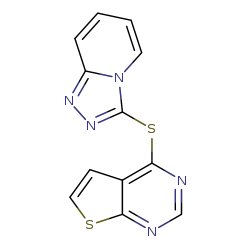 | No | No | Not tested |
| 47A | Enamine, Z31206481 | 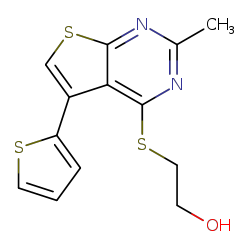 | Yes | Yes | Lower IC_50_ ratio |
| 48A | Enamine, Z31243469 | 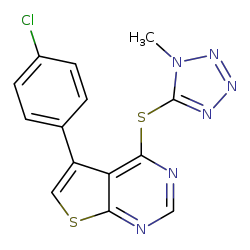 | No | Yes | Lower IC_50_ ratio |
| 49A | Enamine, Z31082948 | 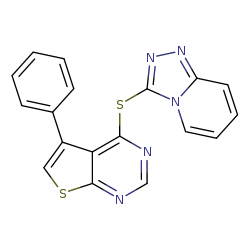 | No | Yes | Lower IC_50_ ratio |
| 50A | Enamine, Z57990683 | 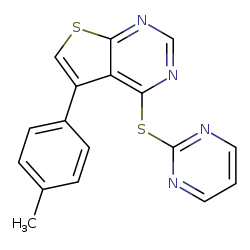 | No | No | Not tested |
| 51A | Enamine, Z2239055447 | 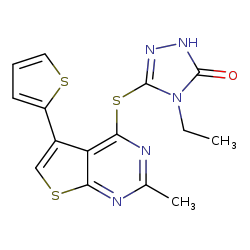 | No | Yes | Lower IC_50_ ratio |
| 52A | Enamine, Z85926207 | 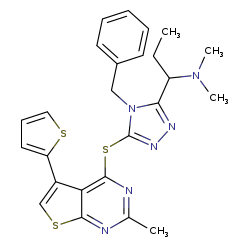 | No | Yes | Lower IC_50_ ratio |
| 53A | Enamine, Z16637984 | 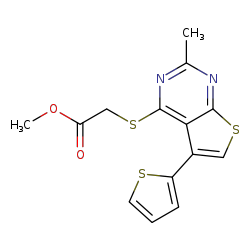 | Yes | No | Not tested |
| 54A | Enamine, Z31243549 | 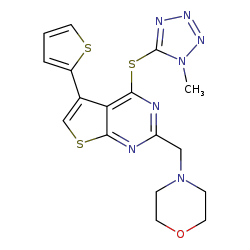 | No | Yes | Lower IC_50_ ratio |
| 55A | Enamine, Z94734908 | 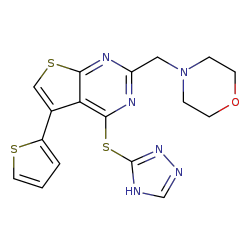 | Yes | No | Not tested |
| 56A | Enamine, Z128124392 | 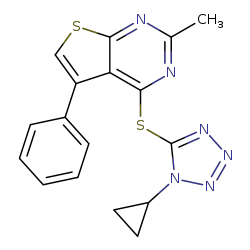 | No | Yes | Lower IC_50_ ratio |
| 57A | Enamine, Z31243468 | 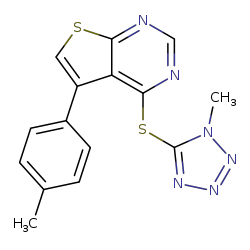 | No | Yes | Lower IC_50_ ratio |
| 58A | Enamine, Z31082949 | 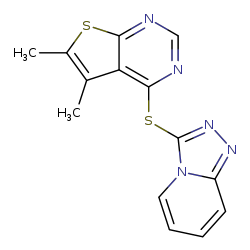 | No | No | Not tested |
| 59A | Enamine, Z56930170 | 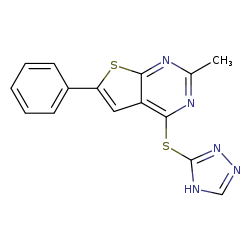 | No | No | Not tested |
| 60A | Enamine, Z31082966 | 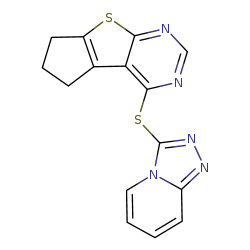 | No | No | Not tested |
| 61A | Enamine, Z128193468 | 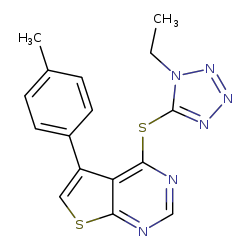 | No | Yes | Lower IC_50_ ratio |
| 62A | Enamine, Z56777524 | 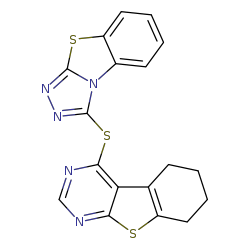 | No | No | Not tested |
| 63A | Enamine, Z198001598 | 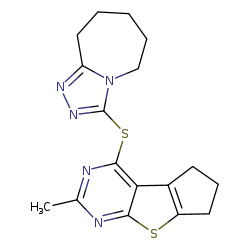 | No | No | Not tested |
| 64A | Enamine, Z198001640 | 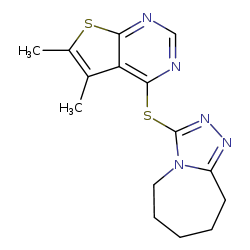 | No | No | Not tested |
| 65A | Enamine, Z198001628 | 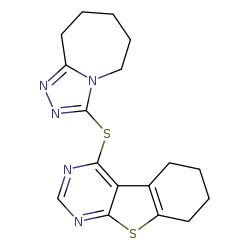 | No | No | Not tested |
| 66A | Enamine, Z275103616 | 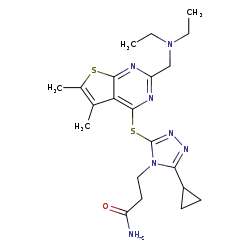 | No | No | Not tested |
| 67A | Enamine, Z2239050295 | 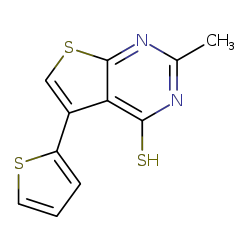 | No | No | Not tested |
| 1B | Enamine, Z16638342 | 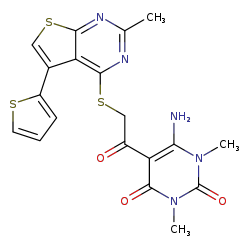 | No | No | Not tested |
| 2B | Enamine, Z26695654 | 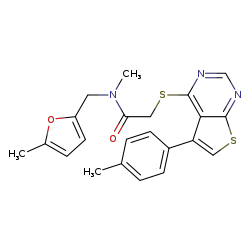 | No | Yes | Lower IC_50_ ratio |
| 3B | Enamine, Z31139179 | 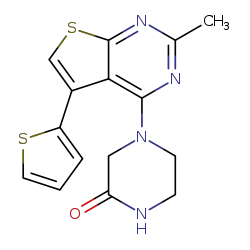 | No | No | Not tested |
| 4B | Enamine, Z103172970 | 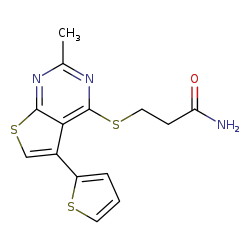 | No | No | Not tested |
| 5B | Enamine, Z16638356 | 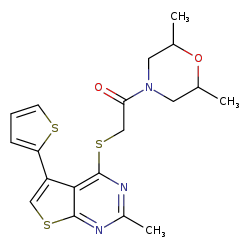 | Yes | No | Not tested |
| 6B | Enamine, Z16637427 | 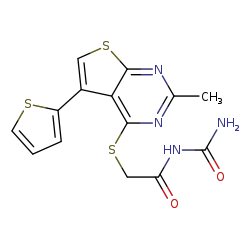 | Yes | Yes | Lower IC_50_ ratio |
| 7B | Enamine, Z16637291 | 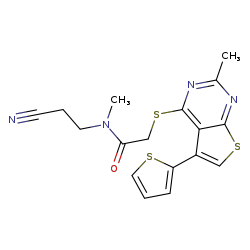 | Yes | No | Not tested |
| 8B | Enamine, Z24279401 | 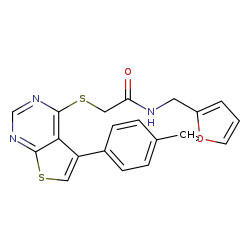 | No | Yes | Lower IC_50_ ratio |
| 9B | Enamine, Z16637398 | 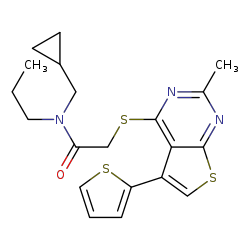 | No | No | Not tested |
| 10B | Enamine, Z52577815 | 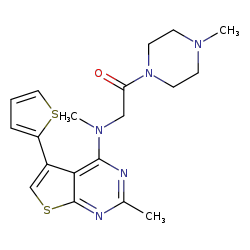 | No | No | Not tested |
| 11B | Enamine, Z20246006 | 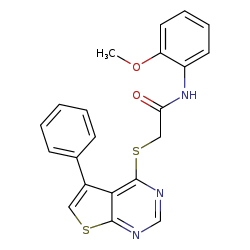 | No | No | Not tested |
| 12B | Enamine, Z56882642 | 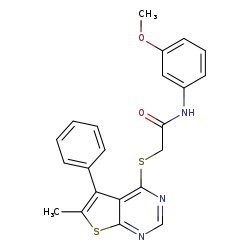 | No | Yes | Lower IC_50_ ratio |
| 13B | Enamine, Z16637043 | 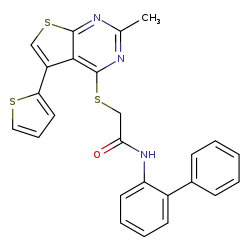 | No | Yes | Higher IC_50_ ratio, but false-positive |
| 14B | Enamine, Z16637598 | 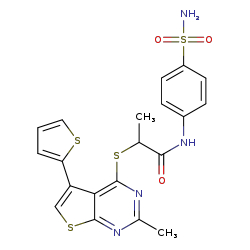 | No | Yes | Lower IC_50_ ratio |
| 15B | Enamine, Z16637300 | 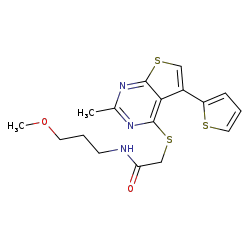 | Yes | No | Not tested |
| 16B | Enamine, Z2241109880 | 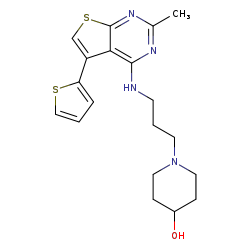 | No | No | Not tested |
| 17B | Enamine, Z16637464 | 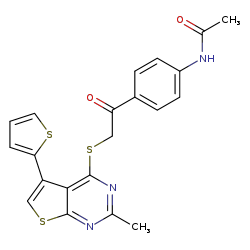 | No | Yes | Higher IC_50_ ratio, but false-positive |
| 18B | Enamine, Z16637803 | 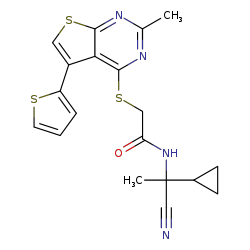 | No | Yes | Higher IC_50_ ratio, but false-positive |
| 19B | Enamine, Z16638051 | 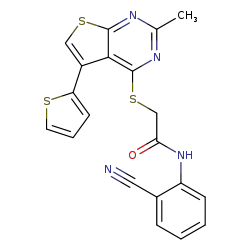 | No | No | Not tested |
| 20B | Enamine, Z24279508 | 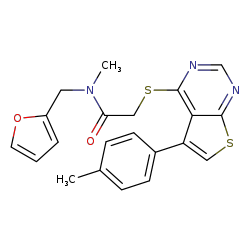 | No | Yes | Lower IC_50_ ratio |
| 21B | Enamine, Z16638047 | 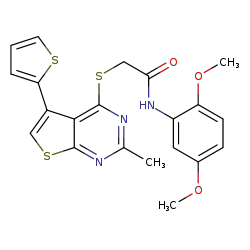 | No | No | Not tested |
| 22B | Enamine, Z16638345 | 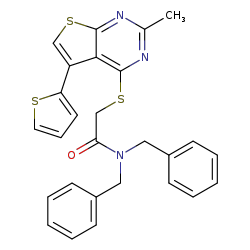 | No | No | Not tested |
| 23B | Enamine, Z128131610 | 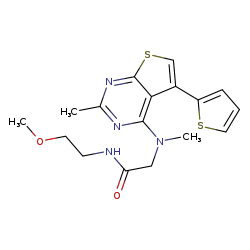 | No | Yes | Lower IC_50_ ratio |
| 24B | Enamine, Z2239074082 | 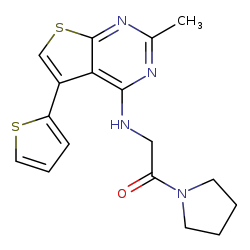 | No | Yes | Lower IC_50_ ratio |
| 25B | Enamine, Z2239055645 | 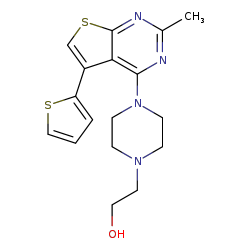 | No | No | Not tested |
| 26B | Enamine, Z16637989 | 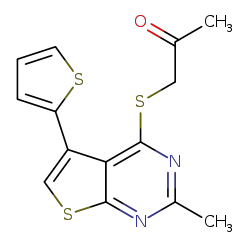 | No | No | Not tested |
| 27B | Enamine, Z16638153 | 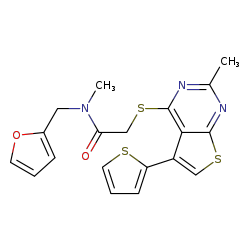 | No | No | Not tested |
| 28B | Enamine, Z16637507 | 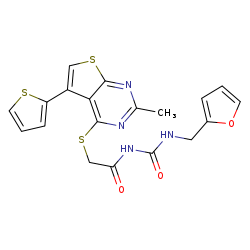 | No | No | Not tested |
| 29B | Enamine, Z16637668 | 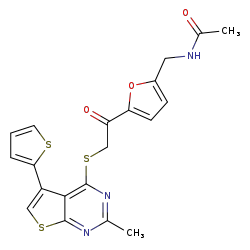 | Yes | No | Not tested |
| 30B | Enamine, Z397859556 | 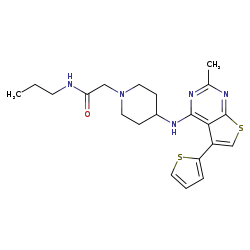 | No | No | Not tested |
| 31B | Enamine, Z16637150 | 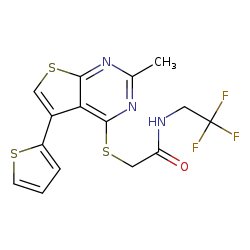 | No | No | Not tested |
| 32B | Enamine, Z16637297 | 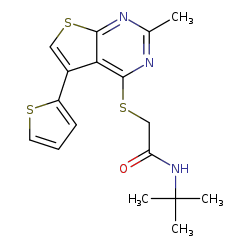 | Yes | Yes | Lower IC_50_ ratio |
| 33B | Enamine, Z66006063 |  | No | Yes | Lower IC_50_ ratio |
| 34B | Enamine, Z16637674 |  | No | No | Not tested |
| 35B | Enamine, Z31245547 |  | No | No | Not tested |
| 36B | Enamine, Z16638028 |  | No | No | Not tested |
| 37B | Enamine, Z31124373 |  | No | No | Not tested |
| 38B | Enamine, Z16637475 |  | No | No | Not tested |
| 39B | Enamine, Z448175334 |  | No | No | Not tested |
| 40B | Enamine, Z20234968 |  | No | Yes | Lower IC_50_ ratio |
| 41B | Enamine, Z16638133 |  | No | No | Not tested |
| 42B | Enamine, Z2239071382 |  | No | No | Not tested |
| 43B | Enamine, Z16637020 |  | No | No | Not tested |
| 44B | Enamine, Z16637285 |  | No | Yes | Lower IC_50_ ratio |
| 45B | Enamine, Z16638041 |  | No | Yes | Higher IC_50_ ratio, but false-positive |
| 46B | Enamine, Z786277332 |  | No | Yes | Lower IC_50_ ratio |
| 47B | Enamine, Z354308096 |  | No | Yes | Lower IC_50_ ratio |
| 48B | Enamine, Z2239076139 |  | No | Yes | Lower IC_50_ ratio |
| 49B | Enamine, Z16637531 |  | No | Yes | Lower IC_50_ ratio |
| 1C | Enamine, Z64573976 |  | No | Yes | Lower IC_50_ ratio |
| 2C | Enamine, Z16637611 |  | Yes | Yes | Lower IC_50_ ratio |
| 3C | Enamine, Z31241653 |  | No | No | Not tested |
| 4C | Enamine, Z128146694 |  | No | Yes | Higher IC_50_ ratio and confirmed |
| 5C | Enamine, Z16638030 |  | No | Yes | Lower IC_50_ ratio |
| 6C | Enamine, Z31109633 |  | No | Yes | Lower IC_50_ ratio |
| 7C | Enamine, Z17142867 |  | No | Yes | Higher IC_50_ ratio, but false-positive |
| 8C | Enamine, Z128193812 |  | No | Yes | Lower IC_50_ ratio |
| 9C | Enamine, Z24279303 |  | No | Yes | Lower IC_50_ ratio |
| 10C | Enamine, Z16636904 |  | Yes | Yes | Lower IC_50_ ratio |
| 11C | Enamine, Z16638360 |  | Yes | Yes | Higher IC_50_ ratio and confirmed |
| 12C | Enamine, Z25464129 |  | No | No | Not tested |
| 13C | Enamine, Z31206475 |  | No | Yes | Lower IC_50_ ratio |
| 14C | Enamine, Z20233718 |  | No | No | Not tested |
| 15C | Enamine, Z2239056480 |  | No | Yes | Higher IC_50_ ratio and confirmed |
| 16C | Enamine, Z200146872 |  | Yes | Yes | Lower IC_50_ ratio |
| 17C | Enamine, Z31206396 |  | No | No | Not tested |
| 18C | Enamine, Z31206484 |  | No | No | Not tested |
| 19C | Enamine, Z16638378 |  | No | No | Not tested |
| 20C | Enamine, Z31085357 |  | No | Yes | Lower IC_50_ ratio |
| 21C | Enamine, Z103173086 |  | No | No | Not tested |
| 22C | Enamine, Z31206398 |  | No | No | Not tested |
| 23C | Enamine, Z31206498 |  | No | Yes | Lower IC_50_ ratio |
| 24C | Enamine, Z31206372 |  | No | No | Not tested |
| 25C | Enamine, Z31147483 |  | No | Yes | Lower IC_50_ ratio |
| 26C | Enamine, Z317301078 |  | Yes | No | Not tested |
| 27C | Enamine, Z31206377 |  | No | No | Not tested |
| 28C | Enamine, Z20235483 |  | No | No | Not tested |
| 29C | Enamine, Z31177327 |  | No | No | Not tested |
| 30C | Enamine, Z31206400 |  | No | No | Not tested |
| 31C | Enamine, Z20231326 |  | No | No | Not tested |
| 32C | Enamine, Z16636927 |  | No | No | Not tested |
| 33C | Enamine, Z1270086424 |  | No | Yes | Lower IC_50_ ratio |
| 34C | Enamine, Z199944968 |  | No | Yes | Lower IC_50_ ratio |
| 35C | Enamine, Z229254086 |  | No | Yes | Lower IC_50_ ratio |
| 36C | Enamine, Z1270086356 |  | No | Yes | Lower IC_50_ ratio |
| 37C | Enamine, Z215012068 |  | No | No | Not tested |
| 38C | Enamine, Z199967676 |  | No | No | Not tested |
| 39C | Enamine, Z246512462 |  | No | No | Not tested |
| 40C | Enamine, Z1478446288 |  | No | Yes | Lower IC_50_ ratio |
| 41C | Enamine, Z20235408 |  | No | Yes | Lower IC_50_ ratio |
| 42C | Enamine, Z1526785534 |  | No | No | Lower IC_50_ ratio |
| 43C | Enamine, Z199784892 |  | Yes | Yes | Not tested |
| 44C | Enamine, Z128124404 |  | No | Yes | Lower IC_50_ ratio |
| 45C | Enamine, Z424820732 |  | No | Yes | Lower IC_50_ ratio |
| 46C | Enamine, Z200177070 |  | No | No | Not tested |
| 47C | Enamine, Z17142572 |  | No | No | Not tested |
| 48C | Enamine, Z2237508497 |  | No | No | Not tested |
| 49C | Enamine, Z20233712 |  | No | No | Not tested |
| 50C | Enamine, Z2241107700 |  | Yes | Yes | Lower IC_50_ ratio |
| 51C | Enamine, Z56770504 |  | No | No | Not tested |
| 52C | Enamine, Z20232029 |  | No | No | Not tested |
| 53C | Enamine, Z25603837 |  | No | No | Not tested |
| 54C | Enamine, Z31120255 |  | Yes | Yes | Lower IC_50_ ratio |
| 55C | Enamine, Z20231985 |  | No | No | Not tested |
| 56C | Enamine, Z31206432 |  | No | No | Not tested |
| 57C | Enamine, Z31206412 |  | No | No | Not tested |
| 58C | Enamine, Z200194736 |  | No | No | Not tested |
| 59C | Enamine, Z25464123 |  | No | No | Not tested |
| 60C | Enamine, Z20245845 |  | No | No | Not tested |
| 61C | Enamine, Z17142544 |  | No | No | Not tested |
| 62C | Enamine, Z2241097168 |  | No | Yes | Lower IC_50_ ratio |
| 63C | Enamine, Z199817466 |  | No | No | Not tested |
| 64C | Enamine, Z64565307 |  | No | Yes | Lower IC_50_ ratio |
| 65C | Enamine, Z20233756 |  | No | No | Not tested |
| 66C | Enamine, Z86389283 |  | No | No | Not tested |
| 67C | Enamine, Z86409302 |  | No | Yes | Lower IC_50_ ratio |
| 68C | Life Chemicals, F5481-2262 |  | No | No | Not tested |
| 69C | Life Chemicals, F5481-0004 |  | No | No | Not tested |
| 70C | Life Chemicals, F5481-0006 |  | No | No | Not tested |
| 71C | Life Chemicals, F5481-0025 |  | No | No | Not tested |
| 72C | Life Chemicals, F5481-0033 |  | No | No | Not tested |
| 73C | Life Chemicals, F5481-0091 |  | No | No | Not tested |
| 74C | Life Chemicals, F5481-0133 |  | No | No | Not tested |
| 75C | Life Chemicals, F5481-0150 |  | No | No | Not tested |
| 76C | Life Chemicals, F5481-2225 |  | No | Yes | Lower IC_50_ ratio |
| 77C | Life Chemicals, F5481-2260 |  | No | No | Not tested |
| 78C | Enamine, Z56882048 |  | No | No | Not tested |
| 79C | LabNetwork, 500194-47-8 |  | No | No | Not tested |
| 80C | LabNetwork, 844449-63-4 |  | No | No | Not tested |
| 81C | LabNetwork, 844652-80-8 |  | No | Yes | Lower IC_50_ ratio |
| 82C | LabNetwork, 332870-43-6 |  | No | No | Not tested |
| 83C | Uorsy, PB1478272140 |  | No | No | Not tested |
| 84C | Uorsy, PB20304241 |  | No | No | Not tested |
| 85C | Uorsy, PB31316924 |  | No | Yes | Higher IC_50_ ratio, but false-positive |
| 86C | Uorsy, PB15764662 |  | No | No | Not tested |
| 87C | Enamine, Z3397472468 |  | Yes | Yes | Lower IC_50_ ratio |
